# Research Progress on Additively Manufactured Porous Structures of Nickel-Based Superalloys

**DOI:** 10.3390/ma19102144

**Published:** 2026-05-20

**Authors:** Shenghang Xu, Yiye Pan, Nanxuan Mei, Shaoqi Jia, Minghao Huang, Chao Ding, Xin Yang, Jinglong Li, Rong Wang, Huiping Tang

**Affiliations:** 1 Advanced Materials Additive Manufacturing Innovation Research Center, Hangzhou City University, Hangzhou 310015, Chinapp1439923693@163.com (Y.P.);; 2 Zhejiang Key Laboratory of Aerospace Metallic Materials, Hangzhou 310015, China; 3 China International Engineering Consulting Corporation, Beijing 100048, China; 4 Defense Innovation Institute, Academy of Military Sciences, Beijing 100071, China

**Keywords:** nickel-based superalloys, additive manufacturing, porous structure, microstructure, properties, applications

## Abstract

Nickel-based superalloys are key materials for aerospace and gas turbine applications. Traditional manufacturing approaches struggle to produce controllable porous structures with complex topologies. This review focuses on additively manufactured porous Ni-based superalloys, and summarizes progress in porous structure design, including disordered, lattice, TPMS, bio-inspired, and AI-assisted structures. Common additive manufacturing technologies are introduced, along with their effects on microstructure evolution and defect formation. The review discusses non-equilibrium microstructures, elemental segregation, and typical defects such as lack-of-fusion, keyhole porosity, and residual stress, as well as their influences on strength, fatigue, and creep behavior. Post-processing strategies for defect mitigation and performance optimization are also summarized. This review highlights the unique mechanical and physical behavior of porous structures compared to bulk materials, with an emphasis on anisotropy, stress localization, and defect sensitivity. Finally, several critical and specific challenges are identified, including multi-scale modeling, microstructure control in complex topologies, fatigue prediction, and physics-constrained AI design. This review aims to provide a clear, focused, and structurally consistent overview of the current state of the field, and to support future research on additively manufactured porous Ni-based superalloys.

## 1. Introduction

Modern industries, especially the aerospace and gas turbine sectors, demand increasingly stringent material performance. To enhance thermal efficiency, turbine inlet temperatures are continuously elevated, imposing higher requirements for the high-temperature mechanical properties and environmental stability of materials. Nickel-based superalloys are hailed as the “heart” of aero-engines, as they retain excellent yield strength, creep resistance, and fatigue resistance, and superior oxidation/hot corrosion resistance at temperatures above 600 °C, coupled with the outstanding thermal and microstructural stability critical for long-term high-temperature service [1,2]. However, traditional dense structures struggle to meet the demands for lightweighting and multifunctional integration under extreme service conditions. The introduction of controllable porous structures into nickel-based superalloys addresses this gap by reducing weight and endowing additional functionalities such as permeability, thermal insulation, energy absorption, and high heat exchange efficiency over large specific surface areas [3].

Traditional manufacturing processes like casting, foaming, and powder metallurgy face limitations in the fabrication of complex porous nickel-based structures. These drawbacks include imprecise control over pore characteristics, poor pore connectivity, and challenges in 3D topological optimization. Additionally, the high hardness of nickel-based superalloys complicates subsequent subtractive machining operations. Additive manufacturing (AM) has fundamentally overcome these constraints, shifting research focus from the “feasibility of fabrication” to “design optimization” and “performance regulation”. The integration of artificial intelligence and machine learning enables performance-oriented inverse design, significantly shortening the R&D cycle of metamaterials [4,5], while post-processing techniques such as heat treatment and hot isostatic pressing (HIP) effectively eliminate internal defects and enhance alloy performance.

However, existing reviews either focus on bulk Ni-based superalloys, summarize general additive manufacturing technologies, or discuss porous structures separately. To date, no review has systematically established a unified process–structure–properties (PSP) framework specifically for additively manufactured porous Ni-based superalloys. Most reviews lack in-depth analysis of the unique thermal behavior, strong anisotropy, stress localization, and defect-dominated failure that distinguish porous structures from bulk materials. Furthermore, few reviews critically evaluate AI-driven inverse design from the perspective of physical consistency and manufacturing constraints. Thus, this review has three distinct and novel objectives:

(1) To fill the research gap with a unified PSP framework for porous Ni-based superalloys, linking melt pool thermal behavior, microstructure, defects, and macro-performance.
(2) To highlight the unique physics of porous structures as an independent material system rather than scaled-down bulk materials.
(3) To provide a critical evaluation of defect mechanisms, fatigue behavior, and AI design, highlighting current bottlenecks and physically consistent future directions.

With this unique synthesis, the review provides a comprehensive, systematic, and forward-looking foundation for the design, manufacture, and engineering application of high-performance porous Ni-based superalloys.

To ensure a rigorous, transparent, and reproducible review process, a systematic literature search strategy was employed. The primary literature was retrieved from databases including Web of Science and Scopus. The search utilized combinations of primary keywords, such as “additive manufacturing” OR “laser powder bed fusion” AND “nickel-based superalloys” (e.g., Inconel 718, Inconel 625, CMSX-4) AND “porous structures” OR “lattice” OR “TPMS” OR “bio-inspired”. To capture the most recent and relevant technological advances, the study selection primarily focused on peer-reviewed journal articles published within the last decade (2015–present), with older foundational papers included for theoretical context. The inclusion criteria strictly required papers to investigate the process–structure–property (PSP) relationships, mechanical performance, or defect mechanisms of AM porous Ni-based superalloys. Studies focusing solely on bulk AM materials without implications for porous architectures, or those lacking sufficient mechanical/microstructural validation, were excluded. This rigorous selection ensured that the synthesis presented herein accurately reflects the most critical and validated advancements in the field.

## 2. Design of Porous Structures

Unlike bulk materials, porous structures fabricated by AM exhibit unique thermal-mechanical characteristics. The surrounding unmelted powder region exhibits extremely low thermal conductivity, resulting in distinct local thermal conditions, slower effective cooling rates, and greater heat accumulation within thin struts. Moreover, the periodic or stochastic geometry of porous structures introduces stronger anisotropy and more severe stress localization compared to bulk materials. Defects, especially those located at nodal joints and thin struts, become far more critical to structural failure, making porous structures an independent material system rather than merely low-density bulk equivalents. It should be noted that when discussing the topological evolution of porous structures (e.g., disordered, lattice, and TPMS), selected examples from non-Ni-based systems (such as SS316L, Ti-6Al-4V, or Fe-35Mn) are periodically cited in this review. These specific examples are strictly selected to illustrate universal structural design principles—such as geometric stiffness limits, macroscopic stress transfer mechanisms, and the elimination of nodal stress concentrations.

### 2.1. Disordered Porous Structures

**Figure 1:**
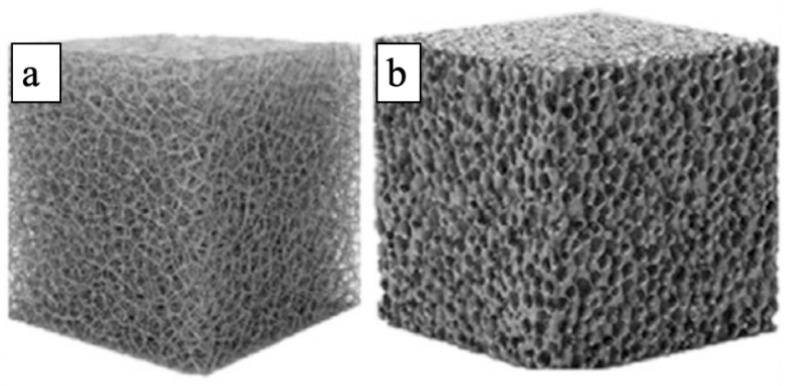
Typical morphologies of (**a**) open-cell and (**b**) closed-cell disordered porous structures [6].

Disordered porous structures have garnered significant attention in materials science and engineering due to their unique macroscopic properties and compatibility with additive manufacturing technologies. Typically mimicking natural structures, such as metal foams or trabecular bone tissues, are characterized by the random distribution of pores and struts [6], as seen in Figure 1, which illustrates the fundamental topological difference between open- and closed-cell porous structures. In the early stages of additive manufacturing, disordered porous structures were primarily generated using stochastic algorithms like Voronoi tessellation, and this random arrangement of internal pores and struts endows them with a certain degree of mechanical isotropy [7]. From the perspective of mechanical behavior, disordered porous structures belong to typical bending-dominated systems, which is a direct consequence of their random topology: the irregularly distributed struts lack directional load-bearing paths, and under external loading, most struts undergo bending deformation rather than axial stretching or compression. This bending-dominated mechanism leads to lower geometric stiffness compared to ordered structures, with the elastic modulus typically following the Gibson–Ashby model [8]:

E = E_o_(*ρ*/*ρ*_o_)^n^

where E_o_ is the elastic modulus of the dense matrix and ρ/ρ_o_ is the relative density, n = 1.5~2.0 for disordered foams.

For the application of nickel-based superalloys, disordered porous structures are often employed in scenarios where the strict uniformity of mechanical properties is not a critical requirement. Instead, the prioritization lies in achieving a high specific surface area or constructing random filtration pathways. For instance, researchers have extensively explored both the functional applications and structural robustness of disordered porous materials. Regarding functional performance, Aldama et al. [9] fabricated disordered nickel foams with hierarchical pores via electrodeposition, leveraging a high specific surface area to significantly increase active sites and facilitate ion diffusion in asymmetric capacitors. Similarly, Luo et al. [9] synthesized disordered lamellar carbon materials using natural zeolite templates, achieving high specific capacitance alongside excellent rate capabilities. However, while these disordered structures enhance electrochemical functionality, they often do so at the expense of mechanical load-bearing capacity; furthermore, their inherent topological randomness makes performance prediction challenging. The energy absorption capacity of disordered porous structures is closely related to relative density and material distribution: as relative density increases, the energy absorption density increases for Ni-based superalloy foams as higher relative density reduces pore size and increases the number of load-bearing struts. However, their random material distribution leads to uneven deformation distribution in the structure volume. The local regions with dense struts bear most of the load and undergo plastic collapse first, while sparse regions exhibit premature buckling, resulting in fluctuating stress–strain curves during compression.

To improve structural controllability, Guo et al. [7] introduced Voronoi diagrams to achieve the statistical regulation of pore parameters via Selective Laser Melting (SLM), revealing the heterogeneous deformation mechanisms of disordered metals during compression. This heterogeneous deformation originates from the combined effect of local elements’ stability and topology. Thick struts in disordered structures tend to undergo plastic collapse due to their high local stability, while thin struts are prone to buckling failure under compression. The random distribution of these struts leads to non-uniform stress transfer paths, with stress concentrated at the junctions of thick and thin struts, which is the main cause of early fracture in disordered porous structures.

While such topological designs enhance predictability, single-phase porous materials still struggle to overcome the fundamental trade-off between high porosity and mechanical strength. Addressing this limitation, Liu et al. [10] proposed the concept of bi-continuous interpenetrating porous composites (BIPCs), integrating ordered lattices with disordered foams through in situ melt foaming and infiltration casting. Their results demonstrated that the synergistic effect between these heterogeneous phases enabled the BIPC to reach a compressive strength of 43 MPa, 4.6 times that of single-phase aluminum foam, and an energy absorption capacity approximately 130% higher than the combined sum of the individual structures. This performance enhancement is attributed to the optimized material distribution and stress transfer: the ordered lattice provides stretching-dominated load-bearing paths to improve geometric stiffness, while the disordered foam fills the gaps to enhance local stability, avoiding premature buckling. The combination of two structural types achieves uniform deformation distribution in the volume, smoothing the stress–strain curve and increasing energy absorption efficiency.

In summary, research on porous structures is transitioning from “random disorder” toward “precision regulation”. Future trends will increasingly focus on leveraging the topological freedom of additive manufacturing, coupled with AI-driven inverse design, to develop multi-scale and multi-phase composite structures that integrate efficient energy/mass transport with exceptional mechanical stability in extreme environments.

### 2.2. Lattice Structures

#### 2.2.1. Truss-Based Lattice Structures

Truss-based lattice structures, composed of nodes and connecting struts, resemble miniature architectural frameworks. Common configurations include body-centered cubic (BCC), face-centered cubic (FCC), and their reinforced variants (e.g., BCC-Z, FCC-Z) [11]. The overall mechanical properties of truss-based lattice structures depend on their topological structure, their relative density, and the properties of the base material used for the struts. According to their deformation behavior, lattice structures can be divided into bending-dominated and stretching-dominated types. Bending-dominated structures mainly dissipate energy through bending, while stretching-dominated structures mainly carry loads through axial tension and compression. For Ni-based superalloys, studies have shown that BCC structures usually have a bending-dominated mode with good flexibility, making them suitable for energy absorption and vibration damping. In contrast, FCC structures are mostly stretching-dominated and show higher specific stiffness and strength, so they are more suitable for load-bearing applications [12].

As a typical truss-based lattice, the BCC structure has been widely investigated. Kokil-Shah et al. [13] fabricated three types of SS316L lattice structures via selective laser melting (SLM), including conventional BCC, BCC with gradient strut diameters, and Z-reinforced BCC (BCCz). Their mechanical responses under quasi-static compression were systematically studied through experiments and finite element analysis. The results demonstrated that topological design plays a key role in mechanical performance. The BCCz structure presented the best load-bearing capacity and energy absorption efficiency, showing a stretching-dominated deformation mechanism and a 62% improvement compared with the conventional BCC. The gradient-reinforced BCC exhibited a moderate enhancement of approximately 22%, while the conventional BCC showed bending-dominated behavior and the lowest overall performance. It was also found that shorter struts contributed to higher yield strength and stiffness, and the mechanical properties were mainly governed by relative density and the number of unit cells. This work provides valuable experimental and numerical references for the structural optimization and engineering application of lattice structures.

**Figure 2:**
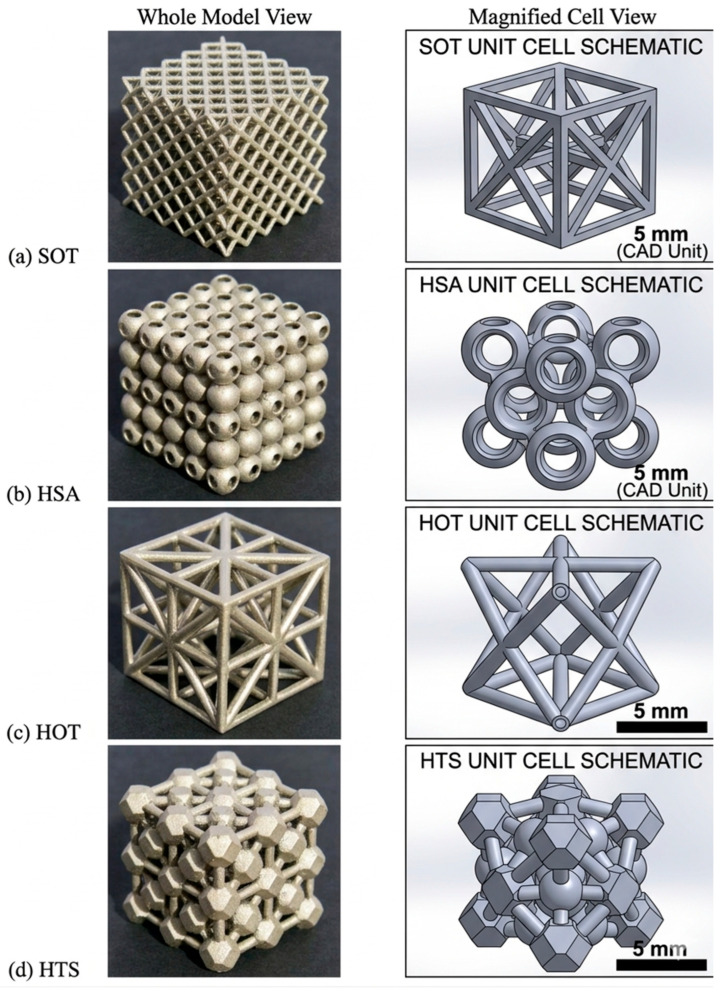
Typical FCC-symmetric lattice structures fabricated by SLM: (**a**) solid octet truss, (**b**) hybrid sphere assembly, (**c**) hollow octet truss, and (**d**) hybrid truss sphere assembly.

In addition, Bonatti et al. [14] investigated the mechanical properties of four FCC-symmetric lattice metamaterials under uniaxial compression, as illustrated in Figure 2, including solid octet truss (SOT), hollow sphere assembly (HSA), hollow octet truss (HOT), and hybrid truss–sphere assembly (HTS). All the samples were fabricated via an SLM of 316L stainless steel, with a relative density of 20%. During compression, the SOT showed localized deformation bands and stress oscillations, similar to conventional foams. In contrast, the other three shell-based structures exhibited stable and uniform macroscopic deformation, with monotonically increasing stress–strain curves. Among them, the HTS structure achieved the highest elastic modulus and energy absorption capacity. The numerical results also suggest that a hollow rhombic dodecahedron shell structure could provide nearly twice the strength and energy absorption of the traditional octet truss.

**Figure 3:**
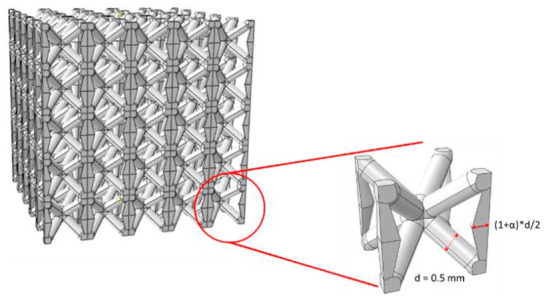
Geometry definition for BCC lattice structures with taper struts [18].

Although AM provides unique advantages for fabricating complex truss-based lattice structures, it still presents notable manufacturing limitations. For example, severe stress concentrations may occur at the nodal regions under loading, which represents one of the dominant causes of failure [15]. To mitigate this issue, Zhao et al. [16] proposed a BCC lattice structure with tapered struts, as illustrated in Figure 3, which demonstrates how tapered struts eliminate stress concentrations at nodal regions, a major source of failure in conventional truss lattices. The results demonstrated that the tapered strut design effectively relocated stress concentrations from the nodes to the middle of the struts, thereby improving the mechanical properties. The failure mode was transformed from nodal shear fracture to combined tensile and plastic failure at the strut center, while the elastic modulus was increased by up to approximately 67% and the structural anisotropy was substantially reduced. In addition, struts with small overhang angles produced by SLM are prone to dross formation and geometric inaccuracies caused by laser energy input and melt pool dynamics, which impair surface quality and dimensional accuracy. Differences between the as-built structure and the design model can further deteriorate the expected mechanical properties. Furthermore, thin struts often undergo deformation during high-temperature heat treatment due to material creep and internal stress relaxation [17]. These issues remain critical challenges for lattice structures that demand high dimensional and geometric accuracy.

#### 2.2.2. Triply Periodic Minimal Surface (TPMS) Structures

TPMS are sheet-based lattice structures that have attracted considerable attention in recent years. As mathematical surfaces with zero mean curvature, TPMS separates space into two interpenetrating but unconnected channels [19]. Common TPMS structures include Gyroid, Diamond, Primitive, and IWP. To clarify the intrinsic origin of performance divergence between different configurations, the core topological and geometric differences in these typical TPMS architectures are defined as follows: (1) Gyroid features a continuous, helical, 3D, interconnected surface with uniform and moderate curvature distribution, no sharp structural transitions, and fully interpenetrating pore channels with consistent tortuosity; (2) Diamond presents a cage-like open topology with larger curved surface units, narrow junction regions between adjacent cages, and discrete high-connectivity pore channels; (3) Primitive has a simple cubic periodic structure with flat surface segments and sharp curved-to-flat transitions at cubic unit edges, forming straight, low-tortuosity pore channels; (4) IWP (Schwarz I-WP) possesses a layered wavy surface with uniform small-scale curvature, highly continuous interlayer pore channels, and a nearly isotropic topological structure. Compared with truss-based lattices, TPMS structures feature continuous and smooth surfaces, which eliminate stress concentrations at nodal regions and thus improve service life and energy absorption efficiency. This structural advantage is further modulated by the intrinsic geometry of each TPMS topology: the uniform continuous curvature of Gyroid and IWP eliminates local stress peaks entirely under both static and dynamic loading; Diamond’s cage-like topology leads to mild stress concentration at the narrow junctions between adjacent surface units; and Primitive’s flat-to-curved transitions create non-uniform stress gradients at cubic unit edges, which become the preferential sites for deformation initiation. Various TPMS architectures exhibit distinct deformation mechanisms and energy absorption characteristics under compressive loading.

**Figure 4:**
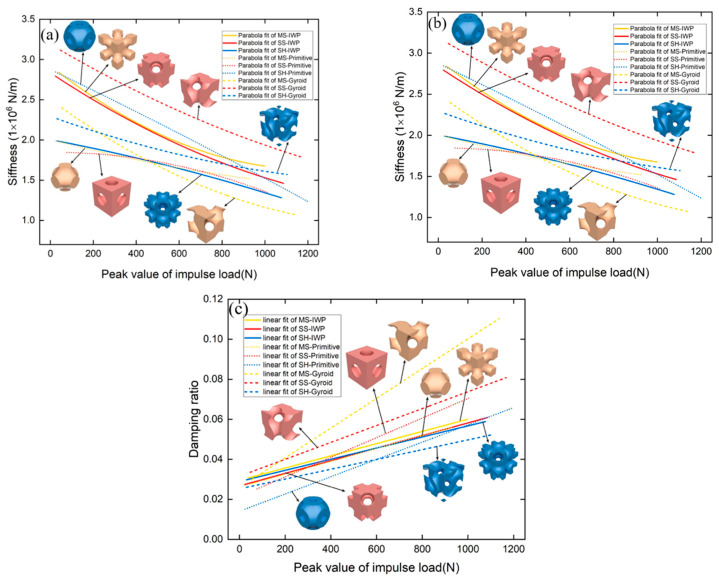
(**a**) Linear fitting of the frequencies; (**b**) parabolic fitting of stiffness values; (**c**) linear fitting of the damping ratios of the different TPMS structures [20].

Fu et al. [20] systematically investigated the nonlinear dynamic behavior of nine TPMS-based structures via impact hammer tests, including three topologies (IWP, Gyroid, Primitive) and their sheet, solid, and pore counterparts, all at a fixed relative density of 50%. As illustrated in Figure 4, the natural frequency, dynamic stiffness, and damping ratio of TPMS structures varied nonlinearly with increasing excitation amplitude. Specifically, natural frequency and dynamic stiffness decreased under elevated loading, revealing a stiffness-softening behavior, while the damping ratio increased, indicating enhanced energy dissipation. Among all structures, the solid-shell Gyroid (SS-Gyroid) exhibited the highest stiffness and damping ratio, delivering the best overall dynamic performance. This work was the first to reveal the damping characteristics and stiffness-softening behavior of TPMS structures under variable-amplitude excitation, providing valuable experimental support for their applications in vibration reduction and lightweight design. In another study, Dargusch et al. [21] fabricated biodegradable Fe-35Mn porous scaffolds via SLM with three TPMS topologies (Gyroid, Diamond, and Schwarz) with three relative densities (42%, 60%, and 72%), and systematically evaluated their mechanical properties, degradation behavior, and cytocompatibility. The Schwarz structure at a relative density of 42% achieved the highest compressive modulus of 25.1 GPa, while the Gyroid structure at the same relative density exhibited the highest yield strength of 77 MPa. These results confirm the regulatory role of relative density and topology on mechanical behavior: as relative density increases, the geometric stiffness and local stability of TPMS structures significantly increase; and for Gyroid structures, yield strength increases linearly with relative density (σ_y_ = σ_y0_(ρ/ρ_0_)^1.3^) due to the enhanced load-bearing capacity of the continuous sheet. The higher yield strength of Gyroid compared to Schwarz is attributed to its more uniform material distribution, which avoids local weak regions and ensures consistent deformation across the structure volume.

Beyond mechanical performance, TPMS structures present distinctive advantages in other fields. Owing to their high surface-to-volume ratio and highly interconnected pore networks, TPMS structures are regarded as promising candidates for high-performance heat exchangers, as they can significantly enhance convective heat transfer efficiency. Studies have shown that thermal performance can be further optimized by controlling the deformation parameter of TPMS architectures [22]. In biomedical applications, TPMS structures morphologically resemble the trabecular bone and possess high porosity, creating a favorable microenvironment for cell adhesion and proliferation, and nutrient transport. Combined with their excellent biocompatibility, TPMS structures have been widely used in the design of bone tissue engineering scaffolds [23].

In AM Ni-based superalloys, complex melt pool dynamics and solidification behavior often result in heterogeneous microstructures. Moreover, removing internal supports from components with intricate flow channels remains highly challenging [24,25]. The integration of TPMS structures into Ni-based alloy flow-channel components effectively addresses these challenges. Owing to their excellent self-supporting properties, TPMS architectures can significantly reduce or even eliminate the need for internal supports. Gyroid and IWP’s continuous curved surfaces with moderate overhang angles exhibit excellent self-supporting capability, eliminating the need for internal supports during fabrication, which avoids the internal defects and performance degradation caused by incomplete support removal. This not only simplifies post-processing and reduces manufacturing costs but also avoids performance degradation or leakage risks caused by incomplete support removal [25]. In contrast, Diamond’s large cage units require partial internal supports at the bottom of the cages, and Primitive’s flat overhang segments have poor self-supporting performance, increasing the difficulty of fabrication and post-processing for Ni-based superalloy components with complex porous structures.

### 2.3. Bio-Inspired Structures

**Figure 5:**
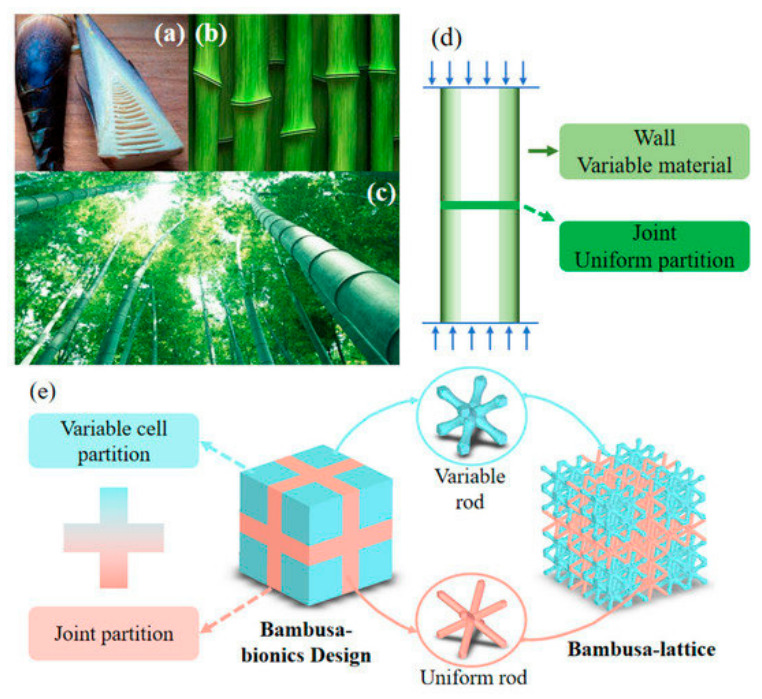
Bambusa lattice structure inspired by bambusa joints and gradient tube walls: (**a**–**d**) bambusa joint and tube structures; and (**e**) bambusa bionic design [31].

Over millions of years of evolution, nature has evolved numerous lightweight, high-strength, and high-efficiency structures. Bioinspired structural design draws on such natural wisdom to provide new strategies for the design of porous Ni-based superalloy structures. As illustrated in Figure 5, Bamboo’s vascular system, which forms the porous structure, comprises interconnected channels and a multi-cell network with a gradient distribution of parenchyma cells. From a mechanical design perspective, bio-inspired structures excel at optimizing material distribution and stress transfer paths, which directly determines their deformation mechanism and energy absorption capacity. Most bio-inspired structures are stretching-dominated or mixed-mode systems, as natural evolution tends to prioritize efficient load-bearing and energy dissipation.

Chouhan et al. [25] fabricated samples via Stereolithography Apparatus (SLA) 3D printing and performed quasi-static compression tests. The results revealed that the bio-inspired spoke structure exhibited the optimal overall performance. The (0.9, 40°) structure achieved a maximum compressive load of 48.023 kN and a compressive strength of 35.214 MPa, outperforming the centriole-inspired structure (16.264 kN, 29.283 MPa) and the nautilus-inspired structure (21.630 kN, 31.018 MPa). At a similar volume, the bio-inspired spoke structure weighed only approximately 1/3 that of a solid cylinder of the same size, while its load-bearing stress was 2.3 times higher, demonstrating the remarkable advantages of bioinspired designs in balancing lightweight characteristics and high mechanical performance. The superior mechanical behavior of the spoke structure is attributed to three key factors: (1) Geometric Stiffness: The radial spokes are aligned along the principal stress direction, forming stretching-dominated load-bearing paths that enhance overall stiffness (E ∝ (ρ/ρ_0_)^1.1^, and are lower than the FCC lattice but higher than BCC. (2) Local Stability: The spoke–node connection adopts curved transitions, avoiding sharp angles and improving the buckling resistance of local elements. (3) Deformation Distribution: The uniform arrangement of spokes ensures that deformation is evenly distributed in the structure volume, avoiding localized stress concentration and premature failure [26]. Finite element analysis further confirmed that bio-inspired geometries regulate stress distribution and failure modes, indicating that such designs can effectively improve the mechanical efficiency and functional applicability of lattice structures.

Nacre is a representative bio-inspired structure mimicking the nacreous layer of shells. Composed of 95% brittle calcium carbonate and 5% organic matrix, this architecture exhibits exceptional strength and toughness, with its fracture toughness being thousands of times higher than that of pure calcium carbonate [27,28,29]. The mechanical enhancement mechanism of nacre-inspired structures lies in their hierarchical material distribution and deformation coordination: the alternating organic–inorganic layers optimize stress transfer, with the organic matrix preventing crack propagation by bridging gaps, while the inorganic layers provide high geometric stiffness. Under compression, nacre-inspired porous structures absorb energy through layer-by-layer plastic deformation and crack bridging, achieving an energy absorption density of 40~50 MJ/m^3^ at 70% relative density—significantly higher than disordered foams and comparable to TPMS structures [30].

In our previous work [32], inspired by the geometric patterns of Chinese knots, we designed and fabricated a lattice structure with 100% material distribution along the load-bearing direction by extruding a two-dimensional planar pattern along the loading direction. The design concept of “full material distribution along the load-bearing direction” proposed in this work offers important insights for the design of lattice structures. By concentrating materials along the primary load-bearing direction, the specific strength can be significantly enhanced without a notable increase in density. From a mechanical perspective, this structure is a typical stretching-dominated system: the material distribution along the load-bearing direction maximizes axial load-bearing capacity, minimizing the bending deformation of structural elements. The high specific strength originates from the optimized geometric stiffness (E ∝ (ρ/ρ_0_)^1.0^) and excellent local stability—no buckling occurs under tensile loading, and failure is dominated by plastic collapse of the load-bearing segments. The uniform material distribution also ensures that deformation is concentrated in the load-bearing direction, avoiding the wasteful deformation of non-load-bearing regions and improving energy absorption efficiency. This load-bearing mechanism is applicable to all lattice structures, providing a basis for the design of Ni-based lattice structures.

### 2.4. AI-Driven “Forward–Inverse” Designed Structures

Forward design aims to predict the macroscopic equivalent properties of lattice structures based on given geometric parameters, including cell size, wall thickness, relative density, and topological configuration. The output properties include effective elastic modulus, ultimate strength, and thermal conductivity [33]. The core goal of this method is to use neural networks (NNs) as efficient surrogate models to replace traditional high-fidelity finite element simulations, thereby reducing the computational resources and time required for design iterations [34]. For example, Bai et al. [35] constructed a prediction model for Young’s modulus and yield strength using nodal coordinates and connectivity matrices as input features. They classified lattice structures into stretching-dominated and bending-dominated deformation modes and trained the model separately, which exhibited high prediction accuracy and significantly higher speed than traditional finite element calculations. However, this model has obvious limitations in generalization: it was trained and validated only on lattice structures with specific topological types (stretching/bending-dominated), and its prediction accuracy drops sharply when extended to complex hybrid-topology structures (e.g., TPMS–truss composite structures) or Ni-based superalloy porous structures with non-equilibrium microstructures, as the training data did not include the influence of material intrinsic properties (e.g., grain size or elemental segregation) on macroscopic performance. Liu et al. [36] established the relationship between lattice geometric parameters and elastic modulus via combined numerical analysis based on the homogenization method. They used Latin hypercube sampling to generate parameter combinations, employed polynomial regression to predict relative density, and then input the predicted relative density as a feature into a random forest model for elastic modulus prediction. The results showed that the prediction error of this model was less than 10% compared with finite element analysis, and it had good robustness against interference from noise. Under the same relative density constraints, the elastic modulus of the optimized lattice structure was increased by up to 25% compared with conventional configurations. Nevertheless, the model’s ability to extrapolate beyond the training data range is limited: the Latin hypercube sampling was conducted within a narrow relative density interval of 40~80%, and when predicting the elastic modulus for porous structures with a relative density below 30% or above 90%, which is critical for transpiration cooling and load-bearing applications of Ni-based superalloys, the prediction error exceeds 30% due to the lack of corresponding training samples and the change in deformation mechanism—for instance, brittle fracture dominates in low-density structures.

In contrast, the inverse design deduces the optimal or feasible lattice configuration based on preset target performance requirements. This is more challenging because multiple structures may have the same macroscopic performance, or slight performance changes may require distinct microstructures [37,38,39]. Peng et al. [40] constructed a bi-material composite triangular lattice structure and generated a dataset containing geometric parameters and equivalent elastic properties based on the computational homogenization method, which was then used to train forward and inverse neural network surrogate models. These models allowed for significant regulation of equivalent elastic properties, for example, the equivalent Young’s modulus and shear modulus could span several orders of magnitude, and the equivalent Poisson’s ratio could be adjusted over a wide range from negative to positive. Furthermore, the parameter error of the structure inverted by the model for the set target performance was less than 5%. However, the model lacks physical consistency constraints: the inverted structures may have geometric features that violate basic manufacturing principles (e.g., a strut diameter smaller than the minimum forming size of SLM for Ni-based superalloys, ~100 μm) or mechanical equilibrium (e.g., local stress concentration exceeding the material yield strength under target loads), as the training process was only optimized for property matching without integrating physical laws (e.g., solid mechanics, additive manufacturing process constraints). Abu-Mualla et al. [41] proposed an inverse design framework based on Physics-Guided Neural Networks (PGNNs) for the performance-oriented design of 3D lattice mechanical metamaterials. They constructed a parameterized dataset containing cubic and anisotropic units and trained a dual-network model by combining physical constraints from numerical homogenization simulations. The model can generate corresponding geometric parameters based on the target stiffness tensor. In terms of computational efficiency, traditional numerical homogenization (MATLAB, R2024a) requires 27.079 s for a single structure, while both forward prediction and inverse reconstruction using the proposed framework take only microseconds. During training, the forward network took 7 min, and the PGNN inverse model took 34 min and 22.5 s. In external cubic dataset tests, the normalized mean square error of 70.37% of the reconstructed structures was below 0.05, and the reconstruction of the entire dataset took only 0.53 s. However, on the Lumpe and Stankovic dataset, only 32% of the reconstruction results met acceptable standards, indicating that the coverage of the current training data remains limited. This limitation reflects the common generalization problem of AI inverse design models: the training dataset is often constructed based on idealized geometric units with uniform material properties, while real additively manufactured Ni-based superalloy porous structures involve complex factors such as non-equilibrium microstructures, pores, residual stress, and topological deviations, which are rarely included in the training data, leading to poor performance when the model is applied to practical engineering scenarios.

**Figure 6:**
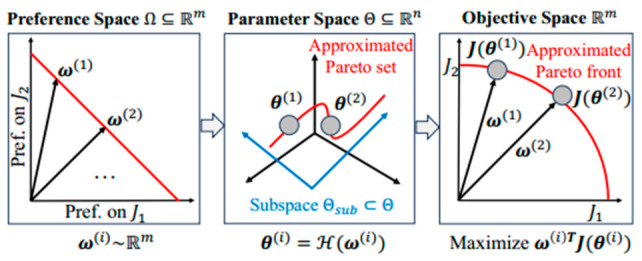
Schematic of the Hyper-MORL framework for approximating the Pareto front in inverse design [42].

Additionally, the inverse design framework can address conflicting objectives (e.g., lightweighting, high strength, and energy absorption) and identify optimal trade-off solutions by plotting Pareto optimal sets, as shown in Figure 6. The multi-objective inverse design logic for balancing lightweight, strength, and energy absorption requirements is shown. By mapping parameter space to objective space, the Pareto set identifies optimal porous topologies. This AI framework enables the performance-oriented design of Ni-based superalloy porous structures while satisfying manufacturing constraints, which effectively improves the physical consistency and engineering feasibility of the design results. Gao et al. [42] successfully designed novel topological configurations with superior anti-buckling performance using a bottom-up strategy combining building blocks and optimization algorithms. The results demonstrate that the buckling strength of the lattice structure achieved via this inverse design method is 30~90% higher than that of conventional diagonal-reinforced grid structures, and 10~30% higher than that of high-performance sponge-inspired designs. Furthermore, the deep neural network prediction model supporting this design flow demonstrated good accuracy: the coefficient of determination (R^2^) for buckling strength prediction reached 0.846 for single angles and averaged 0.952. These quantitative results fully confirm the effectiveness and practicality of this inverse design method in overcoming the limitations of traditional configurations and specifically enhancing the anti-buckling ability of lattice structures. However, similar to other AI-driven design methods, this framework also faces challenges in physical consistency and manufacturing adaptability for Ni-based superalloy porous structures: the designed topological configurations are optimized based on idealized material models, and the influence of AM process parameters on the formability of complex topological features is not considered. As a result, the inverted structures may be difficult to fabricate via SLM/SEBM technologies, or the actual performance after fabrication may deviate significantly from the target due to process-induced defects.

However, most current AI-driven design methods remain overly data-driven and lack physical constraints, making it critical to distinguish transferable and non-transferable approaches for porous Ni-based superalloys. Realistically transferable methods include shallow neural networks, defect-aware surrogate models, and physics-guided neural networks that incorporate manufacturing limits, as these can effectively predict performance while avoiding defects, stress concentrations, and unmanufacturable regions. In contrast, purely data-driven models such as GANs, unconstrained deep networks, and small-sample extrapolation models are not yet transferable, as they tend to generate overly thin struts, sharp nodes, or defect-prone regions that violate the unique thermal, cracking, and forming constraints of Ni-based superalloys. Therefore, only physics-constrained, manufacturing-compatible, and defect-aware AI design frameworks have practical value for this material system.

Beyond data-driven AI design, the digital twin concept offers a systematic framework to achieve the closed-loop, full-chain integration of design, manufacturing, and performance evaluation for porous Ni-based superalloys. A typical digital twin architecture includes multi-physics modeling, real-time in situ monitoring, heterogeneous data fusion, and interactive feedback between virtual models and physical printing processes. At its core lies model–data integration, which combines high-fidelity thermal–mechanical simulations, microstructure evolution models, and data-driven surrogate models with actual process data and experimental measurements. For porous structures with fine struts and complex topologies, digital twin can be used to predict melt pool stability, identify defect-prone regions in nodes and overhangs, dynamically optimize process parameters, and establish a more reliable PSP relationship. In this way, the digital twin acts as a key bridge to connect computational design, additive manufacturing, and service performance evaluation for porous superalloy components.

In summary, AI-driven forward–inverse design has shown great potential in accelerating the development of porous structures. However, its application in additively manufactured Ni-based superalloys still faces three core, critical limitations that require a more balanced and physically grounded perspective: (1) Generalization and the “Black-Box” Nature: Most data-driven models act as “black boxes” trained on highly specific, limited topological types like simple cubic lattices and specific material datasets. They fundamentally lack the mechanistic understanding required to generalize to complex hybrid topologies such as graded TPMS or to account for the non-equilibrium microstructures unique to AM Ni-based superalloys. (2) Extrapolation Beyond Training Data: A severe bottleneck of purely data-driven AI is its inability to extrapolate. The prediction and design accuracy degrades sharply—often producing mathematically absurd topologies—when the target performance parameters (e.g., ultra-low relative density < 10% or high density > 90%) fall outside the interpolation boundaries of the training data. This limits their reliability in extreme environment applications. (3) Absence of Physical Constraints: Many existing AI models optimize geometry solely based on mathematical objective functions, entirely ignoring governing physical constraints. This absence of embedded physics and AM process limitations frequently results in “optimal” designs that are theoretically superior but practically unmanufacturable or physically unstable. Future frameworks must transition from purely data-driven to physics-informed neural networks (PINNs) to ensure structural stability and manufacturability.

### 2.5. Quantitative Mechanistic Comparison of Typical Porous Structures

The macroscopic mechanical behavior of porous Ni-based superalloys is fundamentally governed by the topological architecture, which determines the intrinsic stress transfer path, geometric stiffness efficiency, structural stability, and deformation uniformity. To clarify the intrinsic performance differences among disordered porous structures, truss-based lattice structures, TPMS structures, and bio-inspired structures, a quantitative mechanistic comparison is systematically conducted from four core physical dimensions, addressing the transition from structural description to underlying physical principles:

(1) Stress Transfer Mechanism: Stress transfer in disordered structures relies on highly random load paths, leading to severe localized stress peaks. In contrast, truss-based lattice structures offer defined stress transfer paths. However, their sharp geometric transitions induce distinct stress localization at the nodal joints. Quantitatively, the theoretical stress concentration factor (*K_t_*) at these nodes can exceed 3.0, leading them to act as primary crack initiation sites during fatigue loading. TPMS structures fundamentally resolve this nodal weakness through their zero-mean-curvature topologies, facilitating smooth stress distribution with *K_t_* values approaching ideal unity (*K_t_* ≈ 1.0~1.2). Meanwhile, bio-inspired structures exhibit hierarchical stress transfer mechanisms, dynamically redistributing stress through multi-scale features.
(2) Geometric Stiffness: Geometric stiffness, characterized by the elastic modulus and specific strength at the same relative density, directly reflects the lightweight load-bearing efficiency of porous architectures, which can be quantitatively characterized by the scaling exponent n in the Gibson–Ashby model (E = E_0_(ρ/ρ_0_)^n^). Disordered porous structures are typical bending-dominated systems with a scaling exponent n = 1.5~2.0 [8]. Truss-based lattice structures exhibit topology-dependent transfer modes: conventional BCC lattices are bending-dominated with n = 1.8~2.0, while reinforced BCCz, FCC, and octet–truss lattices transform into stretching-dominated systems with n reduced to 1.0~1.2, where the load is borne by axial tension/compression of struts, achieving a 62% improvement in load-bearing capacity compared with conventional BCC [13]. TPMS structures are typical stretching-dominated systems with n = 0.8~1.1, where the continuous smooth curved surface realizes uniform load transfer through membrane stress, completely eliminating the stress concentration caused by discontinuous nodes in truss lattices [20]. Additionally, bio-inspired structures exhibit hierarchical stress transfer mechanisms, dynamically redistributing stress through multi-scale features. At a fixed relative density of 20%, disordered porous structures exhibit the lowest geometric stiffness, with an elastic modulus only 30~40% of that of stretching-dominated truss lattices and 20~25% of that of high-performance TPMS structures. For truss-based lattices, the tapered strut BCC design increases the elastic modulus by up to 67% compared with the conventional equal-diameter BCC, while the HTS shell-based FCC structure achieves nearly twice the strength of the traditional solid octet truss [14,16]. For TPMS structures at 70% porosity and 250 °C, the IWP configuration achieves a yield strength of 225.76 MPa, which is 48.1% higher than that of the Diamond structure of 152.42 MPa, 63.4% higher than that of the Gyroid structure (138.18 MPa), and 80.7% higher than that of the Primitive structure (124.95 MPa) [26].
(3) Local Structural Stability: The local structural stability determines the failure mode and critical bearing capacity of porous structures, which is governed by the topological continuity, stress distribution, and geometric transition characteristics of the architecture. In disordered structures, thin struts are highly prone to Euler buckling under low critical loads [7]. For truss-based lattice structures, the conventional equal-diameter strut design has severe stress concentration at nodal regions, which is the dominant origin of failure. The tapered strut design effectively relocates stress concentration from the nodes to the middle of the struts, transforming the failure mode from nodal shear fracture to combined tensile and plastic failure at the strut center, and substantially reducing structural anisotropy [16]. The shell-based FCC structures (HSA, HOT, HTS) avoid localized deformation bands compared with solid octet trusses, showing more stable deformation behavior [14]. TPMS structures exhibit the best local stability: the continuous smooth surfaces of Gyroid and IWP completely eliminate local stress peaks under static and dynamic loading, with no sharp structural transitions. The IWP structure avoids the 45° shear band instability common in Diamond structures, while the Primitive structure has non-uniform stress gradients at the cubic unit edges, which become the preferential sites for deformation initiation [20,26]. Bio-inspired structures leverage energy absorption mechanisms (e.g., interlocking elements) to arrest localized buckling before it triggers global collapse.
(4) Deformation Distribution: The uniformity of deformation distribution directly affects the energy absorption efficiency, stress–strain curve stability, and fatigue performance of porous structures under compressive loading. Disordered porous structures show extremely uneven deformation distribution. This random topology leads to uneven load transfer paths, where local regions with dense struts bear most of the load and undergo plastic collapse first, while sparse regions exhibit premature buckling, resulting in significant fluctuations in the stress–strain curve during compression [7]. Truss-based lattice structures exhibit a topology-dependent deformation distribution: conventional BCC lattices show localized deformation concentrated at nodal regions, with progressive layer-by-layer collapse and obvious stress oscillations. The reinforced BCCz and tapered strut BCC lattices achieve more uniform deformation transferred to the strut midsection. The shell-based FCC structures (HSA, HOT, HTS) exhibit stable and uniform macroscopic deformation with monotonically increasing stress–strain curves, without localized deformation bands [13,14]. TPMS structures show the most uniform deformation distribution: IWP and Gyroid structures present a typical layer-by-layer plastic collapse mode without localized shear bands or deformation concentration. The Diamond structures are prone to localized deformation along 45° shear bands, while Primitive structures show preferential deformation initiation at the flat–curved transition edges of cubic units, with inferior deformation uniformity compared with Gyroid and IWP [20,26]. Bio-inspired structures enable a controlled, step-wise progressive deformation behavior, dissipating energy through sequential layer collapse rather than catastrophic shear band formation.

To provide clear guidance for structural design in AM Ni-based superalloys, a global comparative synthesis contrasting these four structural classes and their underlying physical mechanisms is summarized in Table 1.

## 3. Classification and AM Technologies of Ni-Based Superalloys

As the core process level in the PSP framework, the selection of AM technologies and process parameters for Ni-based superalloys directly determines the thermal history of the molten pool during the fabrication of porous structures. Different AM technologies have distinct thermal characteristics, and the adjustment of process parameters further modulates the thermal history of the molten pool. These thermal characteristics are the fundamental driving force for the evolution of microstructure and defects in the structure level of the PSP framework, and also the key factor that restricts the formability of porous structures.

**(a)**  **Classification of Ni-based Superalloys for AM**

The AM processability of Ni-based superalloys is closely related to their chemical composition, especially the content of γ′-phase-forming elements such as Al and Ti. According to their cracking susceptibility, Ni-based superalloys can be classified into two categories: solid-solution-strengthened alloys with good weldability, and precipitation-strengthened alloys that exhibit poor weldability.

Solid-solution-strengthened alloys (e.g., Inconel 625, GH3536, Hastelloy X) generally contain very low or no γ′ phase. Their strengthening mainly relies on lattice distortion caused by solute atoms (e.g., Cr, Mo, Fe) in the matrix, which impedes dislocation motion [43,44,45]. Since a very low or no γ′ phase is formed, their solidification path is close to the eutectic type with a relatively narrow solidification temperature range, leading to better processability and lower cracking susceptibility during SLM. Their crack resistance can be further improved by introducing reinforcing phases into the alloy [46,47]. Ranjbar et al. [47] fabricated a Hastelloy X superalloy via SLM and achieved a relative density of 99.8%. It was found that the addition of 1 wt.% CeO_2_ particles can eliminate hot cracking and evidently refines the grain structure. In a 3.5% NaCl solution at 70 °C, the CeO_2_-reinforced Hastelloy X superalloy exhibited excellent corrosion resistance, with a polarization resistance of 85.6 kΩ∙cm^2^ and a passivation range of approximately 1078 mV.

Precipitation-strengthened alloys (e.g., Inconel 718, Inconel 738, CM247LC, K438) contain high Al and Ti contents and achieve outstanding high-temperature strength through the formation of a large-volume fraction of γ′ phase [48,49,50,51]. However, this makes them highly prone to various cracks during the SLM process, which severely degrade the structural integrity and mechanical performance of the as-built components. For such alloys, current research strategies mainly focus on three aspects. First, the substrate is preheated to above 500 °C to reduce thermal stress. Second, the alloy composition is changed by reducing the C and Si contents and adding grain boundary-strengthening elements, such as B, Zr, and Hf. Third, specialized scanning strategies need to be developed [46,52,53].

**(b)**  **Selective Laser Melting (SLM)**

**Figure 7:**
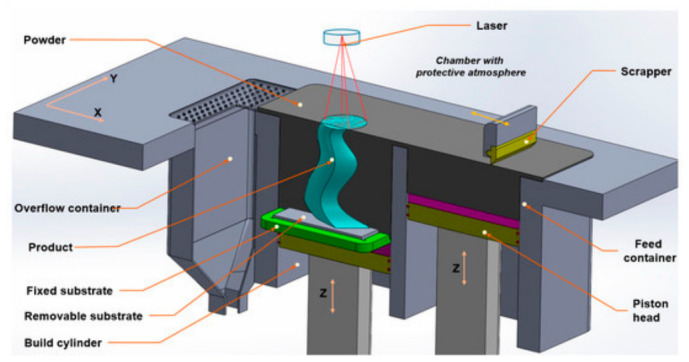
Schematic of the laser powder bed fusion (SLM) process [55].

SLM is currently the most widely used AM technology for fabricating high-precision, high-density Ni-based superalloy components, with its technical principle illustrated in Figure 7. This technology uses a high-energy laser beam as the heat source. Under a protective inert atmosphere, the pre-placed metal powder is selectively melted layer by layer according to cross-sectional slicing data, and metallurgical bonding is formed via rapid melting and solidification [54]. For Ni-based superalloys, SLM is extensively applied to solid-solution-strengthened alloys with low crack susceptibility, such as Inconel 718 and Inconel 625.

For porous structures, thin struts are surrounded by low-conductivity powder, leading to significantly different thermal conditions compared to bulk samples. Heat dissipation is hindered, cooling rates are reduced, and heat accumulation becomes prominent. These unique thermal environments alter solidification conditions, segregation behavior, and phase precipitation, which cannot be predicted using bulk material criteria.

**Figure 8:**
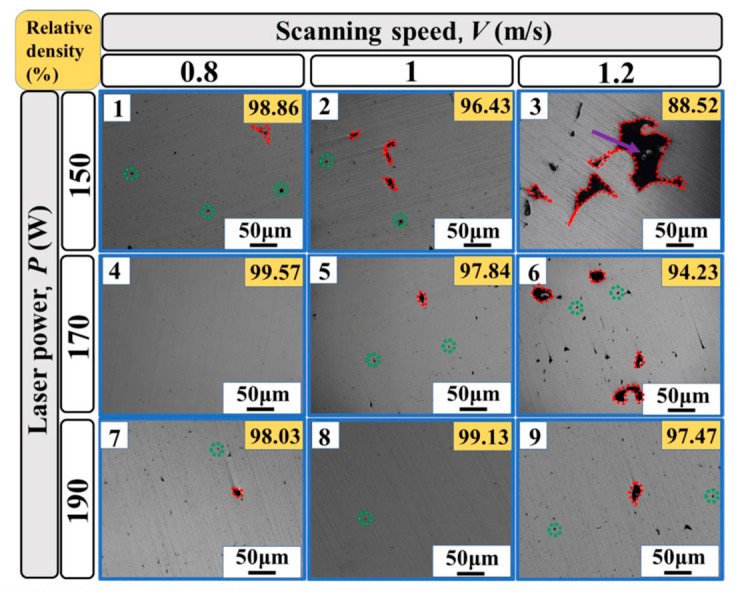
OM images of SLMed GH99 samples with different SLM parameters [56].

To precisely regulate the fabrication quality of porous structures, it is essential to establish a quantitative correlation between process parameters and defect formation. Figure 8 illustrates a representative additive manufacturing process window, typically defined by laser power (P) and scanning speed (V), which systematically delineates the stable processing regime from various defect zones. However, dedicated process window data for nickel-based alloys tailored to porous structures is not yet available, and most existing windows are developed for fully dense components. At insufficient energy densities, lack-of-fusion (LoF) voids tend to form due to poor wettability or incomplete powder melting. Conversely, excessive energy input triggers a transition into the “keyhole” mode, leading to the entrapment of shielding gases or metallic vapors. Furthermore, high scanning speeds often induce melt pool instabilities, such as the “balling” effect, which compromises the continuity and dimensional accuracy of micro-struts. The construction of such process windows facilitates the identification of a viable manufacturability envelope, providing a theoretical foundation for the precise fabrication of high-performance porous architectures.

**(c)**  **Selective Electron Beam Melting (SEBM)**

**Figure 9:**
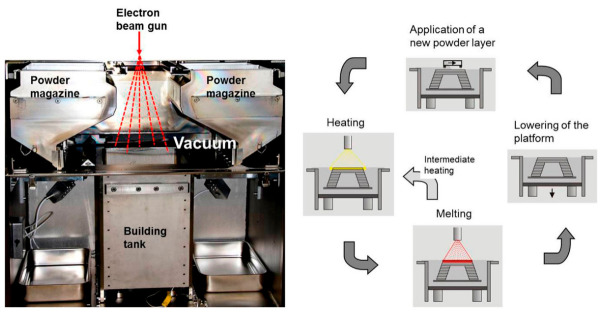
Schematic of the selective electron beam melting (SEBM) process [59].

Figure 9 shows the working principle of SEBM, which uses an electron beam as the heat source to melt metal powder in a vacuum environment. Compared with SLM, SEBM generally enables a higher build temperature and allows for fabrication under elevated preheating temperatures. This high preheating temperature reduces thermal gradients and residual stresses, thereby suppressing the formation of hot cracks. For high-γ′-phase alloys such as Inconel 738, which are prone to cracking during SLM, the high-temperature preheating of SEBM keeps the material above its brittle transition temperature. This significantly reduces thermal stress accumulation and effectively inhibits the initiation of cold cracks and strain-age cracks [57]. Li et al. [58] established a defect-free criterion with defect sizes below 50 μm and no cracks or linear defects. Based on this criterion, they determined the process window for an IN738LC alloy fabricated by SEBM, with an energy density of 4.2–7.0 J/mm^2^. Under these conditions, samples with fine γ′ precipitates of 111~266 nm were directly fabricated. Their high-temperature mechanical properties were significantly superior to those of the as-cast material. Specifically, a tensile strength of 177 MPa and an elongation of 24% were achieved at 1100 °C.

Furthermore, the SEBM process enables exceptional control over the microstructure. Because the electron beam can flexibly regulate the local heat flow direction, temperature gradient, and growth rate, SEBM can maintain the precise solidification conditions required for single-crystal growth during fabrication. This enables single-crystal texture control comparable to directional solidification casting, which is extremely difficult to achieve using laser-based additive manufacturing [60]. Chauvet et al. [61] successfully fabricated crack-free single-crystal specimens of a non-weldable Ni-based superalloy analogous to CMSX-4 via SEBM. Experiments were performed within a linear energy range of 0.3–0.6 J/mm. Specifically, employing a high linear energy of 0.6 J/mm produced a melt pool depth of approximately 532 μm and a primary dendrite spacing of about 30 μm, corresponding to a cooling rate of ~30 °C/s. After reaching a build height of approximately 10 mm, competitive grain growth induced the gradual evolution of columnar grains with a single [001] orientation into a monolithic single-crystal structure, which was confirmed to be a fully dense, crack-free single crystal by Electron Backscatter Diffraction (EBSD). This study demonstrated that precise control of process parameters promotes columnar grain growth and intensifies grain competition, thus enabling single-crystal fabrication without grain selectors or seed crystals.

**(d)**  **Directed Energy Deposition (DED)**

**Figure 10:**
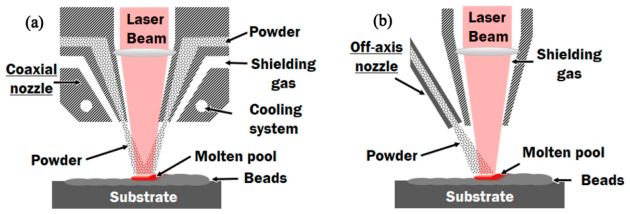
Schematics of (**a**) coaxial and (**b**) off-axis powder feeding for laser directed energy deposition. [69].

DED is another advanced AM process characterized by synchronous material feeding and in situ melting, whose schematic diagram is illustrated in Figure 10. Raw materials in powder or wire form are synchronously delivered into a molten pool generated by a high-energy beam, where they undergo rapid melting and subsequent solidification to achieve layer-by-layer material deposition and metallurgical bonding [62]. In contrast to SLM, a unique characteristic of DED lies in its coaxial powder-feeding nozzle, which precisely converges the powder into the focal region of the high-energy beam. This distinctive design endows DED with several distinct advantages, including the freedom from build space constraints, the ability to repair damaged components, and the feasibility of fabricating functionally graded materials [63].

**Figure 11:**
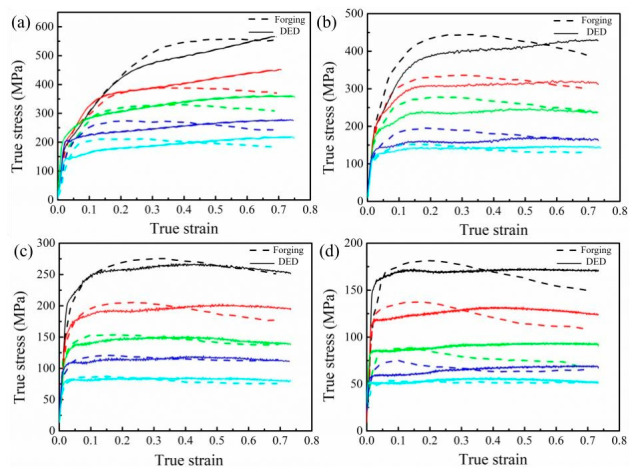
True stress–true strain curves of DED and forged Inconel 625: (**a**) strain rate of 1 s^−1^; (**b**) strain rate of 0.1 s^−1^; (**c**) strain rate of 0.01 s^−1^; (**d**) strain rate of 0.001 s^−1^ [67].

The Inconel 625 Ni-based superalloy is widely employed in DED additive manufacturing due to its outstanding corrosion resistance, high-temperature strength, and good processability, especially for surface cladding to improve component performance [64,65]. By precisely controlling process parameters, DED allows for regulation of the microstructure of Inconel 625 superalloy, thereby achieving a high-quality metallurgically bonded interface [66]. Furthermore, as shown in Figure 11, the research has revealed that the Inconel 625 superalloy fabricated by DED exhibits higher flow stress during hot compression testing than wrought Inconel 625, and its dynamic recrystallization behavior is sensitive to deformation temperature and strain rate [67]. In addition, Haynes 230 demonstrates excellent forming stability in DED. Muhammad et al. [68] successfully fabricated a Haynes 230 Ni-based superalloy via DED, and the as-deposited superalloy achieved a yield strength of 433 MPa and an elongation of 36~38%, showing favorable comprehensive mechanical properties. After heat treatment at 900 °C, the strength of the as-deposited superalloy was largely preserved at 431 MPa, while solid-solution treatment at 1177 °C not only maintained high strength but also further improved the elongation of the alloy. These results indicate that the Haynes 230 Ni-based superalloy is highly compatible with the DED process, which can directly produce an as-deposited microstructure with balanced strength and ductility, and its performance can be further optimized via post-deposition heat treatments.

Furthermore, owing to its distinctive real-time powder mixing capability, the DED technology exhibits unique advantages in the fabrication of functionally graded materials and metal matrix composites, thereby enabling the customization of material properties and the integration of multiple functions. Song et al. [70] utilized DED technology to regulate the content of W addition, ranging from 0 to 16.5 wt.%. The results indicated that W addition significantly enhanced the mechanical properties of the Inconel 625 alloy. With the addition of 6.0 wt.%, the elongation reached 51.8%, corresponding to an approximate 38% increase compared to the base alloy. At a W addition of 16.5 wt.%, the tensile strength attained 1167 MPa, representing an increase of approximately 289 MPa.

**(e)**  **Wire Arc Additive Manufacturing (WAAM)**

**Figure 12:**
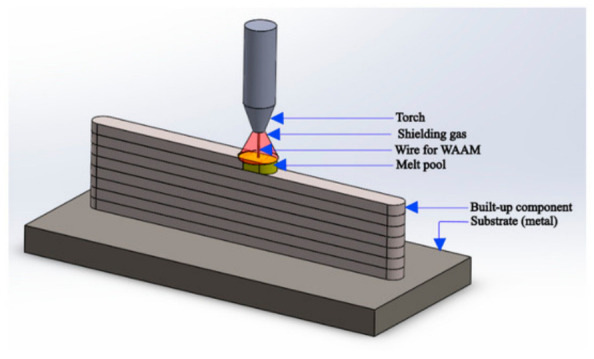
Schematic of the wire arc additive manufacturing (WAAM) process [77].

WAAM employs an electric arc as the heat source to melt metallic wire, fabricating components via layer-by-layer deposition, whose schematic is illustrated in Figure 12 [71]. Compared with other AM techniques, WAAM presents a remarkable advantage in deposition rate, enabling the cost-effective and efficient production of large-scale metallic components for aerospace and energy industries [72]. For Ni-based superalloy porous structures, WAAM’s unique process characteristics determine its distinct PSP correlation: the high heat input (10^3^~10^4^ J/mm, 10~100 times higher than SLM) leads to a low cooling rate (10~100 K/s), which directly induces coarse columnar grains (grain size > 100 μm) and severe elemental segregation (Nb enrichment up to 8 wt.% in interdendritic regions) in porous structures—this is in sharp contrast to the fine grains (grain size < 10 μm) and moderate segregation of SLM-fabricated samples [69,71].

WAAM exhibits great potential in fabricating large-scale Ni-based alloy structural parts, especially for components such as exhaust cones and flanges, applied in the aerospace and energy industries [73,74]. However, the inherently high heat input characteristic of the WAAM process induces severe metallurgical defects in as-fabricated parts, mainly including coarse grains and elemental segregation, which lead to significant anisotropy in the components. Quantitatively, the tensile strength of WAAM-fabricated Inconel 718 porous structures along the build direction (BD) is 420~480 MPa, while it decreases by 15~25% to 340~400 MPa perpendicular to BD and the elongation shows a similar anisotropic trend [71]. This anisotropy originates from the preferential growth of columnar grains along BD and the directional distribution of Laves phase in interdendritic regions, which act as brittle fracture paths under transverse loading [72]. Therefore, WAAM-fabricated Ni-based alloys generally require a combination of interpass cold working and rigorous homogenization heat treatment to refine grains and eliminate such defects [75,76].

To address these issues and optimize the PSP correlation for WAAM Ni-based porous structures, two key technical paths have been validated: (1) Interpass Cold Working (e.g., rolling with 10~15% reduction): This process introduces plastic deformation to break coarse columnar grains, promoting the recrystallization of fine equiaxed grains (grain size reduced to 20~30 μm) and reducing elemental segregation by enhancing solid-state diffusion—after treatment, the tensile strength anisotropy is reduced to <8%, and the average strength is increased by 10~15% [72]. (2) Double-Stage Homogenization Heat Treatment (1150 °C/4 h + 1050 °C/2 h): This treatment dissolves the coarse Laves phase formed during WAAM, releases Nb solute into the γ matrix, and promotes the uniform precipitation of the γ″ strengthening phase (particle size ~50 nm) during subsequent aging—compared with as-deposited samples, the yield strength is increased by 30~40% (from 320 MPa to 420~450 MPa) and the ductility is improved by 20~25% [71].

For porous structure fabrication, WAAM’s high deposition rate makes it suitable for the large-scale open-cell porous structures (porosity 30~60%, pore size 500~2000 μm) used in gas turbine exhaust systems and industrial high-temperature flue gas filtration. However, its limited forming precision of ±0.5~1.0 mm restricts its application in fine porous structures. Future research should focus on developing wire feeding optimization strategies (e.g., variable wire feed rate) to control pore size distribution and reduce segregation, and integrating in situ rolling with WAAM to realize simultaneous forming and grain refinement, thereby expanding its application scope in medium-precision large-scale Ni-based porous components [68,73].

**(f)**  **Binder Jetting (BJ)**

**Figure 13:**
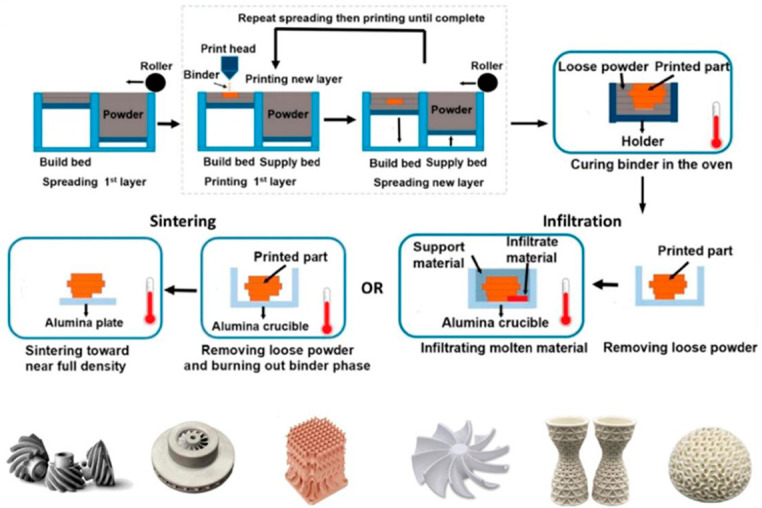
Schematic of the binder jetting (BJ) additive manufacturing system [82].

BJ is a typical powder-bed-based AM process, and a precision printhead is used in this process. According to sliced layer information, the printhead selectively delivers liquid binder onto a powder bed that has been uniformly spread in advance, and this liquid binder then binds the powder particles together to form an integral green part. A schematic diagram of this process is shown in Figure 13. The green part is then subjected to debinding to remove the organic binder, followed by high-temperature vacuum sintering near the alloy solidus temperature, where densification is achieved through solid/liquid phase diffusion. A distinctive characteristic of BJ is that no melting of metallic powder occurs throughout the entire fabrication process—this eliminates the rapid heating–cooling cycles inherent in SLM/SEBM, thereby avoiding thermal stress and cracking [78,79,80]. For Ni-based superalloys with high cracking susceptibility, this is a critical advantage, as it enables the fabrication of complex porous structures that are difficult to achieve via laser-based technologies [77]. Thus, the BJ method completely eliminates the rapid heating–cooling cycles inherent in SLM, thereby avoiding thermal stress and cracking. This makes it particularly suitable for fabricating Ni-based superalloy components with extremely complex geometries, thin walls, and high susceptibility to deformation. In addition, the BJ process can be employed to prepare metal matrix composites such as Inconel 718/TiC. Owing to the absence of intense melting, the reinforcing TiC particles can be uniformly distributed within the matrix and form a well-bonded interface via reactive sintering, which significantly enhances the wear resistance and hardness of the material. This also avoids the agglomeration or decomposition of ceramic particles commonly observed in laser-based melting processes [81]. Quantitatively, BJ-fabricated Inconel 718/TiC (5 wt.% TiC) porous structures (porosity 40%) exhibit a Vickers hardness of 380~420 HV, which is 40~50% higher than that of pure Inconel 718 porous structures (260~280 HV), and the wear rate is reduced by 60~70% (from 8.5 × 10^−6^ mm^3^/(N∙m) to 2.5~3.0 × 10^−6^ mm^3^/(N∙m)) [74].

The pore structure of BJ Ni-based porous structures is primarily regulated by two key process parameters: (1) Powder Size Distribution: Using a bimodal powder (coarse powder 50~100 μm + fine powder 10~20 μm) optimizes the powder bed packing density, leading to a more uniform pore size distribution and higher porosity controllability [75]. (2) Sintering Temperature: Increasing the sintering temperature from 1250 °C to 1320 °C promotes atomic diffusion, increasing the relative density of porous structures from 85% to 95%, but reduces porosity by 8~10%—the trade-off between densification and porosity must be balanced based on application requirements [76].

For engineering applications, BJ’s low residual stress and high shape complexity make it ideal for fabricating porous Ni-based components such as gas turbine combustor liners and nuclear reactor liquid metal filters. However, its low sintering density and slow fabrication speed are major limitations. Future research should focus on developing high-efficiency debinding–sintering integrated processes to reduce fabrication time, and optimizing powder modification to improve sintering activity and achieve full densification while maintaining target porosity [78].

**(g)**  **The Process Window for Porous Structures**

While robust process windows mapping laser power against scan speed are well-established for dense, bulk AM Ni-based superalloys, a consolidated, universally accepted process-window data set specifically derived for AM porous Ni-based superalloys is currently absent from the open literature. This absence represents a critical, unresolved research gap. The existing literature often relies on empirical trial-and-error derived from bulk specimens, which fundamentally fails when applied to micro-scale bounding geometries.

**Figure 14:**
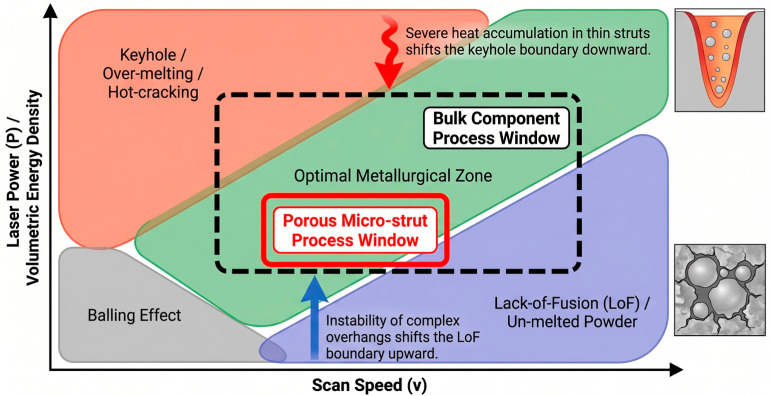
Conceptual process-window and defect-map schematic for AM Ni-based superalloys.

To conceptualize this severe limitation and connect AM parameter sensitivity with manufacturability, Figure 14 illustrates a schematic defect map comparing the theoretical process windows of bulk vs. porous AM Ni-based superalloys. In the low-energy-density regime, the LoF boundary shifts upwards for porous components, as thin struts with diverse overhang angles require precise energy input to ensure inter-layer bonding without causing excessive melt pool collapse. Conversely, in the high-energy-density regime, the threshold for keyholing and hot-cracking is drastically lowered. The restricted heat dissipation in thin struts triggers severe thermal accumulation, pushing the melt pool prematurely into the keyhole mode and inducing liquation cracking. Consequently, the safe “optimal processing window” for AM porous Ni-based superalloys is significantly contracted compared to bulk materials. This dictates that the utilization of static process parameters is inherently flawed for complex porous topologies, firmly underscoring the future necessity of geometry-adaptive, in situ dynamic parameter control.

In conclusion, the above AM technologies and process parameters form the diverse process regulation system in the PSP framework for Ni-based superalloy porous structures, and the thermal history induced by different processes directly leads to the differentiation of the subsequent microstructure and defects. The selection of AM technologies and process parameters for Ni-based superalloys dictates the thermal history of the melt pool during porous structure fabrication, which further governs microstructure evolution, defect formation, and formability within the PSP framework. The process window is highly sensitive to laser power, scanning speed, and layer thickness: an inappropriate process window leads to unstable melting, insufficient fusion, balling, spattering, and thermal cracking. Defect maps reveal that thin struts, overhangs, and internal porous regions are particularly vulnerable to defects and residual stress concentration. Melt pool stability determines the feasibility of fabricating thin struts, while practical fabrication limits constrain the minimum strut diameter, minimum overhang angle, and maximum complexity of porous geometries. Therefore, achieving controllable, defect-free, and repeatable manufacturing requires a comprehensive consideration of process window sensitivity, melt pool stability, defect formation, and practical fabrication limits. Thus, the following chapter will focus on the structure level of the PSP framework and elaborate on the evolution law of non-equilibrium microstructures and typical defects of AM Ni-based superalloy porous structures. Additionally, post-processing strategies such as the PSP regulation bridge will be systematically introduced to optimize the microstructure and defects, thereby realizing a targeted improvement in macroscopic properties.

## 4. Microstructural Evolution and Post-Processing

### 4.1. Formability of AMed Porous Structures

As the interface link between process and structure in the PSP framework, the formability of AMed porous structures is jointly determined by the AM process parameters and porous structure topological design. The formability not only reflects the matching degree of process parameters and structural design but also directly determines the initial microstructure and defect state of the as-fabricated porous structures, which is the foundation of the subsequent microstructure evolution and performance formation in the PSP framework.

As the strut diameter decreases, the thermal environment around porous struts differs drastically from bulk materials because of the low thermal conductivity of the surrounding powder bed. Heat cannot be dissipated efficiently, resulting in higher thermal gradients, more significant residual stress, and easier balling or sagging. This means that process parameters optimized for bulk materials cannot be directly applied to porous structures. Therein, SLM is a common method for fabricating porous Ni-based superalloys, where process parameters play a crucial role in the formability of the porous structures. Specifically, laser power and scanning speed together determine the energy density input into the powder bed. Excessive energy input can induce the keyhole effect, resulting in irregular pores, while insufficient energy may lead to lack-of-fusion defects inside the structure and an increase in porosity. Additionally, by controlling the pulse characteristics of the laser, fine control over the pore size of the porous structure can be achieved, and it is even possible to fabricate microporous structures with feature sizes far below the traditional minimum for SLM. For instance, Jafar et al. [83] successfully fabricated stainless steel porous structures with significantly reduced pore sizes via a pulsed-wave mode SLM technique. Their findings revealed that by adjusting the laser power and defocusing distance, the average pore radius could be controlled within the range of 9~23 μm while maintaining a relatively high porosity of 2~42%, which is much lower than the typical pore sizes of 50~150 μm achieved by traditional SLM processes. This case is emphasized to illustrate a universal mechanism applicable to most AM porous Ni-based superalloys.

Additionally, different scanning strategies also affect heat accumulation and melt pool stability, thereby influencing the formation of defects and the uniformity of porous structures. When fabricating Ni-based alloys via SLM, the dimensional forming limit for single-strut structures is typically in the range of 100 to 200 μm [84]. Specifically, when the designed diameter falls below 150 μm, the stability of the melt pool decreases significantly, which easily induces the “balling effect” and further leads to strut discontinuity or fracture. Studies have shown that by optimizing the single-track scanning strategy, the feature size can be reduced to ~100 μm. However, the mechanical properties of these structures fluctuate substantially due to geometric irregularities [85].

Typically, the actual as-built dimensions of porous structures are often larger than their designed values. This is because partially melted powder particles tend to adhere to the solid surface at the strut boundaries, a phenomenon that is particularly severe for horizontal or low-angle overhang struts, resulting in reduced pore sizes and deviations in porosity. Specifically, overhang regions lack effective support from the underlying powder, leading to poor heat dissipation and decreasing the stability of the melt pool. Therefore, it is easier for partially melted or spattered powder to adhere to the downward-facing surfaces, thereby causing the actual as-built dimensions to exceed the designed values and increasing surface roughness. Feng et al. [86] investigated the formation mechanism of surface roughness on overhang structures fabricated by SLM and clearly identified overhang angle as a critical factor affecting the surface accuracy of parts. Their findings showed that when the overhang angle is less than 15°, its impact on surface roughness is minimal. When the overhang angle ranges from 15° to 50°, the powder adhesion intensifies due to melt pool sinking and an increased contact area with the powder bed. When the overhang angle exceeds 50°, surface roughness increases sharply, which is attributed to the combined effects of the fluctuations in track profile, powder agglomerate adhesion, severe warping deformation, and the formation of dross. This finding provides a key theoretical basis for controlling the surface quality of unsupported overhang features, which are widely present in porous structures. Yang et al. [87] further demonstrated that pre-polishing combined with micro-arc oxidation (MAO) can effectively improve the surface roughness of SLM components, showing particularly significant optimization effects on high-roughness SLM formed parts, providing a technical reference for the surface quality control of overhang surfaces in porous structures.

### 4.2. Non-Equilibrium Microstructure

The non-equilibrium microstructure is the core structural characteristic in the PSP framework of AM Ni-based superalloy porous structures, and its formation is directly driven by the extreme thermal history induced by AM process parameters. The topological design of porous structures further modulates the distribution of non-equilibrium microstructures, and the non-equilibrium microstructure is the fundamental factor that causes the subsequent formation of defects and the degradation of macroscopic properties in the PSP framework. The unique non-equilibrium microstructures in AMed porous Ni-based superalloys primarily result from melt pool dynamics and the extremely high cooling rates of 10^3^~10^8^ K/s [88]. According to the thermodynamic and kinetic fundamentals of γ′ and γ″ phase precipitation, both γ′ (Ni_3_(Al, Ti)) and γ″ (Ni_3_Nb) strengthening phases are typical diffusion-controlled precipitates, whose nucleation and growth require the long-range diffusion of solute elements (Al, Ti, Nb) in the Ni matrix, and only occur within a specific temperature window (700–950 °C for γ″, 750–1000 °C for γ′ in Inconel 718) with sufficient atomic mobility. Under such rapid cooling conditions, equilibrium strengthening phases, such as γ′ and γ″ phases, which would normally form through diffusion at high temperatures, cannot precipitate in time during the solidification process. This suppression of precipitation can be explained by two core kinetic effects: (1) the solid–liquid interface advances at an ultra-high speed during rapid solidification, leaving no time for solute elements to diffuse and redistribute according to the equilibrium partition coefficient, resulting in the trapping of γ′/γ″-forming elements (Al, Ti, Nb) in the primary γ phase; (2) the melt pool cools from the solidus temperature (~1300 °C) to room temperature within microseconds, passing through the precipitation temperature window of γ′ and γ″ at a rate far exceeding the critical cooling rate required for nucleation. Even if the matrix is thermodynamically supersaturated, the atomic diffusion rate at low temperatures is too low to overcome the critical nucleation energy barrier for precipitation. Consequently, Ni-based superalloys fabricated by SLM form a supersaturated γ matrix [89].

In Ni-based porous structures, the segregation behavior is more significantly affected by the cooling rate: at fine-scaled struts, heat struggles to dissipate through the surrounding powder bed, leading to significant heat accumulation inside the struts. Experimental data shows that compared with structures with a diameter of 10 mm, struts with a diameter of 1 mm have a relatively lower cooling rate, which results in the Laves phase formed in the interdendritic regions becoming coarser and distributed in an elongated shape [90]. The thermodynamic mechanism of elemental segregation and Laves phase formation during non-equilibrium solidification is interpreted as follows: for Ni-based superalloys, the equilibrium partition coefficient k_o_ (the ratio of solute concentration in the solid phase to that in the liquid phase at the solid–liquid interface) of Nb, Mo, and Si is far less than 1 (k_o_ ≈ 0.48 for Nb in the Ni matrix), meaning these elements are preferentially rejected into the residual liquid phase during solidification. According to the Scheil–Gulliver non-equilibrium solidification model, under rapid solidification conditions with negligible solid-state diffusion, solute elements are continuously enriched in the interdendritic residual liquid and the Nb concentration in the liquid phase can increase from the nominal 5 wt.% to over 20 wt.% at the final stage of solidification. Thermodynamically, the enrichment of Nb significantly reduces the Gibbs free energy of the system to form the topologically close-packed (TCP) Laves phase (Ni_2_Nb), providing sufficient driving force for the non-equilibrium eutectic reaction L → γ + Laves, which is suppressed under equilibrium solidification conditions. The lower cooling rate in fine struts prolongs the solidification time, allowing for more sufficient solute diffusion and Nb enrichment in interdendritic regions, which promotes the growth of the Laves phase from a fine granular to coarse elongated morphology. As a brittle, topologically close-packed phase, the Laves phase consumes a significant amount of Nb elements, which suppresses the subsequent precipitation of the γ″ strengthening phase and thereby deteriorates the material properties. This effect can be further explained by its influence on phase stability: the formation of the Laves phase depletes the Nb solute in the γ matrix, reducing the supersaturation degree of Nb and thus lowering the thermodynamic driving force for γ″ precipitation during subsequent heat treatment. Meanwhile, the coarse elongated Laves phase acts as a stress concentration site under loading, promoting crack initiation and propagation, and further reducing the structural stability of the alloy. In addition, Ren et al. [91] thoroughly investigated the dynamic solute transport and elemental segregation during AM using a coupled fluid dynamics and microstructure model, and they confirmed the similar non-equilibrium state induced by the rapid cooling rate.

**Figure 15:**
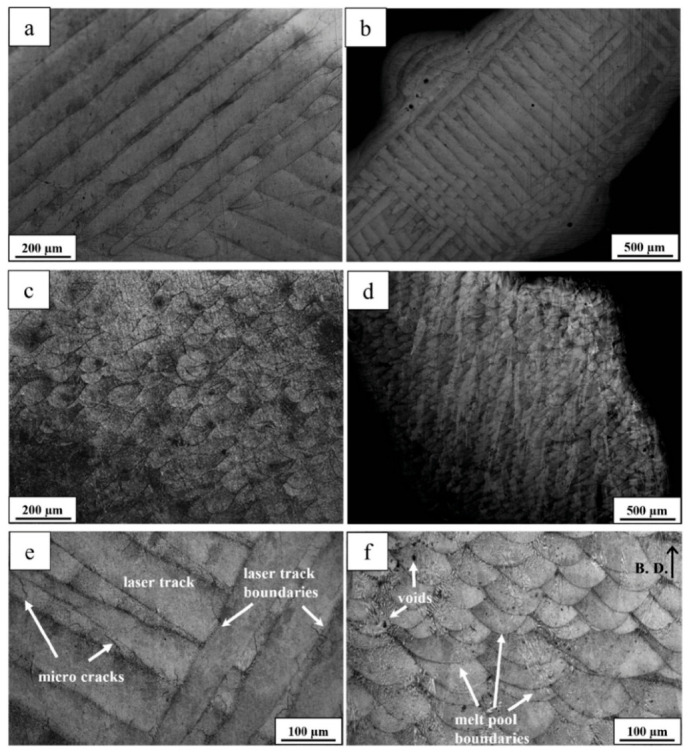
Optical microscope micrographs of IN718 bulk samples and lattice structure A on the xy side (**a**,**b**) and on xz side (**c**,**d**), respectively. Representative microstructural features for the bulk sample are also shown (**e**,**f**). B. D. indicates the building direction [96].

**Figure 16:**
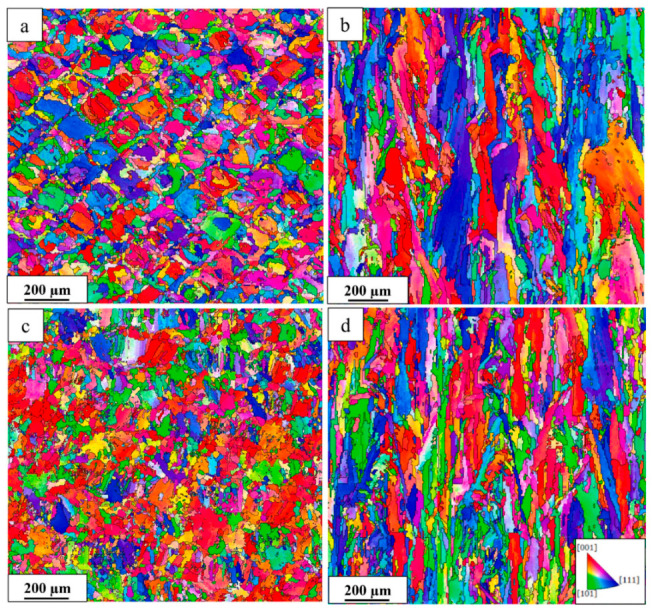
EBSD grain orientation maps of IN718 bulk samples along the z-axis, which corresponds to the building direction, shown in (**a**,**b**) for the xy side and xz side, respectively. In (**c**,**d**), the maps refer to the xy and xz sides, respectively [96].

During the AM process, layer-by-layer melting and rapid cooling lead to epitaxial growth, where the solidified grains at the bottom of the melt pool serve as nucleation sites for the new liquid, resulting in the formation of fine columnar grains aligned along the heat flow direction. For Ni-based alloys with a face-centered cubic lattice structure in Inconel 718, Hastelloy X [75,90], the <001> crystallographic direction is the preferred growth orientation. When the heat flow direction coincides with or is close to the <001> direction, these grains gain an advantage in competitive growth, enabling them to penetrate multiple layers and grow continuously, ultimately forming a columnar grain structure with an extremely high aspect ratio [92]. Compared with bulk materials, porous structures exhibit significantly stronger anisotropy. The slender struts in porous structures are surrounded by unmelted powder with extremely low thermal conductivity, which confines heat transfer along the build direction and creates a pronounced “thermal channel” effect. This unidirectional heat extraction promotes the formation of long, through-penetrating columnar grains along the strut length. As a result, the mechanical properties of porous struts become highly orientation-dependent, a behavior far more intense than that in bulk materials processed under identical conditions. As illustrated in Figure 15 and Figure 16, such strong anisotropy is a distinctive characteristic of porous structures. Because the laser scanning path is limited to only one or a few tracks within narrow struts, the heat flow direction becomes highly singular. Combined with the insulating powder environment, this further strengthens the directional grain growth. Consequently, porous structures cannot be regarded as scaled-down bulk materials, but represent a distinct material system with independent structure-property physics [88].

**Figure 17:**
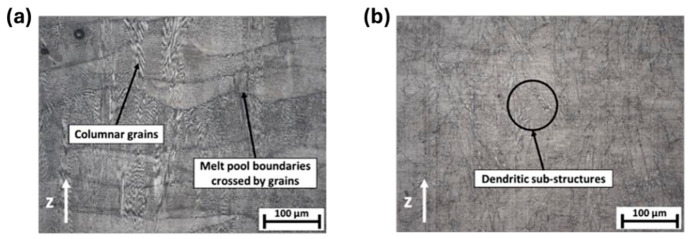
Optical micrographs of the upper plate (YZ plane) of samples: (**a**) C2G-AB, displaying epitaxial grain growth across melt pool boundaries; and (**b**) C2G-HT, showing columnar grains formed after heat treatment with partially retained dendritic substructures [93].

**Figure 18:**
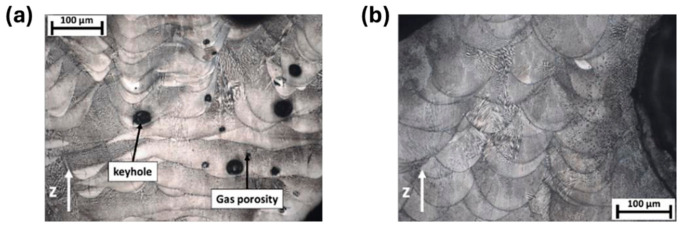
Micrographs showing defects in terms of the porosity observed along the YZ plane in a region of the lattice structure of the samples (**a**) C2F-AB and (**b**) C2G-AB [93].

In contrast, for sheet-based porous structures, such as TPMS, the continuous variation in surface curvature causes the heat flow direction to shift constantly in space, which disrupts the continuity of columnar grains to a certain extent and results in a more random grain orientation, as shown in Figure 17 [93]. The research indicates that the microstructural uniformity of the Gyroid structure is superior to that of strut-based structures, as seen in Figure 18. Its local strain energy distribution is more balanced, which helps to reduce the cracking tendency during the forming process. Furthermore, on the free surfaces of porous units, the melt pool is driven by gravity and surface tension, so the grain morphology is often affected by melt pool surface fluctuations, exhibiting cellular grain characteristics [94,95].

### 4.3. Defect Evolution

In porous structures, defects are far more critical than in bulk materials. Geometric discontinuities at nodes and thin struts induce severe stress localization. Even small defects (pores, lack-of-fusion, or segregation) can act as dominant crack initiation sites, leading to catastrophic failure. In bulk materials, such defects often have minimal influence on overall performance. This difference confirms that porous structures are not just low-density versions of bulk alloys, but a fundamentally different structural system.

To provide practical guidance for quality control in AM porous Ni-based superalloys, it is essential to establish a systematic hierarchy that prioritizes the impact of various defects across different macroscopic failure modes. Defects in porous structures do not contribute equally to failure; rather, their criticality is strictly governed by the loading conditions:

(1) Fatigue Failure (Highest Sensitivity to Sharp Defects): Under cyclic loading, porous structures exhibit extreme sensitivity to geometric discontinuities. In this context, sharp LoF defects and severe surface roughness represent the highest-priority threats. Unlike spherical pores, the sharp tips of LoF defects, coupled with the inherent stress localization at thin struts, generate massive stress intensity factors (ΔK), bypassing the crack initiation phase and leading to rapid, catastrophic fatigue crack propagation.
(2) Creep Deformation (High Sensitivity to Internal and Interfacial Defects): For Ni-based superalloys operating at elevated temperatures, creep resistance is paramount. Here, the hierarchy of critical defects shifts. Internal gas porosity, microcracks, and the elemental segregation at grain boundaries become the dominant life-limiting factors. Under sustained thermo-mechanical loading, internal pores act as nucleation sites for creep cavitation, while segregated brittle phases (e.g., Laves phases in Inconel 718) promote premature grain boundary sliding and intergranular fracture, severely degrading the high-temperature creep rupture life.
(3) Static Strength and Yielding (Sensitivity to Macro-Geometric Deviations): Compared to fatigue and creep, static compressive or tensile strength is relatively defect-tolerant regarding micro-porosity. However, it is highly sensitive to macro-geometric deviations, such as missing struts, strut waviness, or large-scale LoF. These macroscopic defects directly reduce the effective load-bearing cross-sectional area and induce bending moments in theoretically stretch-dominated struts, leading to premature localized yielding, Euler buckling, and a significant drop in overall structural strength and stiffness.

To systematically summarize this hierarchy, Table 2 provides a prioritization matrix correlating specific defect types with their most heavily impacted failure modes and the underlying physical mechanisms.

#### 4.3.1. Residual Stress

During the AM process of porous Ni-based superalloy structure, the residual stress generated through the SLM process still primarily follows the temperature gradient mechanism [97]. However, porous structures present unique localized characteristics. The magnitude and distribution of residual stress in porous structures are remarkably different from bulk materials. The low thermal conductivity of surrounding powder leads to non-uniform temperature fields. Meanwhile, the low stiffness of thin struts magnifies thermal deformation and stress localization. Therefore, residual stress in porous structures cannot be estimated using models developed for bulk materials. The laser or electron beam rapidly heats the fine struts and thin-walled nodes, producing extremely high instantaneous temperatures. In contrast, the surrounding unheated pore boundaries and thick supporting struts stay at relatively low temperatures. This creates a steep temperature gradient within tiny feature sizes [98]. As the newly melted and solidified material layer contracts under high temperatures, it is constrained by the underlying porous skeleton, which has a lower temperature and greater rigidity, generating tensile strain [99]. Since Ni-based superalloys have low yield strength at high temperatures, irreversible plastic deformation occurs when the tensile stress exceeds the yield strength at local thin walls or nodes. After cooling to room temperature, the plastic deformation is retained in the micro-components of the porous structure, ultimately forming a self-balancing residual stress field within the overall framework. This stress field is often highly concentrated in slender struts and areas with abrupt geometric changes, directly affecting the fatigue life and dimensional stability of the porous structure [100]. Additionally, the unique architecture of the porous structure further promotes the generation of residual stress because the pores reduce both thermal conductivity and mechanical constraint, resulting in more non-uniform temperature distributions and cooling rates, and thus increasing the heterogeneity of residual stress [101]. For thin-walled porous structures with low stiffness, this internal stress is fatal. Once cut from the substrate, stress release causes macroscopic warping deformation and can even induce “delayed cracking” during storage [92].

Furthermore, at extremely high temperatures, the material also exhibits creep behavior [102]. However, the ultra-fast cooling rate of additive manufacturing often leads to insufficient creep relaxation at high temperatures, consequently freezing a large amount of residual stress in the material.

#### 4.3.2. Elemental Segregation

AM features extremely rapid melting and cooling rates, which provide insufficient time for solute diffusion. During solidification, solute atoms are preferentially partitioned at the solid–liquid interface, forming a continuous composition gradient from the grain core to the boundary. As a result, severe elemental segregation occurs in the interdendritic or cellular regions [103]. Aina et al. [104] experimentally demonstrated that the high thermal gradient and rapid solidification of Ni-based superalloys during the SLM process lead to obvious interdendritic segregation in the as-built microstructure. The concentration of Nb increases by up to 61% compared with the nominal composition. Such segregation enhances the thermodynamic stability of the δ phase, raising its optimal formation temperature from approximately 950 °C to about 1000 °C. Consequently, after directly applying a standard heat treatment, the volume fraction of the δ phase reaches approximately 11%, which is much higher than the 4.3% obtained in the wrought alloy. The results show that redundant δ phase reduces the ductility and deteriorates the high-temperature elongation and creep rupture life [103].

For porous structures, the adverse effects of elemental segregation become more prominent. Segregation embrittles grain boundaries and reduces creep resistance. At the corners of pores or the transition regions of pore walls, segregation easily induces local stress concentration and the precipitation of brittle phases, which thereby act as initiation sites for microcracks [105]. Li et al. [106] fabricated 304L-Inconel 718 bimetallic materials via laser powder bed fusion. It was found that cracks mainly appeared in the compositional transition zones with a 304L content of 45~75 wt.%. The fundamental reason for this is the severe segregation of solute elements such as Nb at grain boundaries, forming continuous stripe-like brittle NbFe_2_ Laves phases. Under solidification shrinkage stress, these brittle phases trigger the nucleation and propagation of cracks, which seriously restrict the mechanical properties and forming quality of porous structures.

#### 4.3.3. Porosity Defects

In porous structures, defects located at nodes or thin struts have a decisive effect on failure, whereas in bulk materials, similar defects may be harmless. The combined effect of stress localization and thin geometry makes defects far more critical in porous structures, which is a key characteristic distinguishing them from bulk materials.

**Figure 19:**
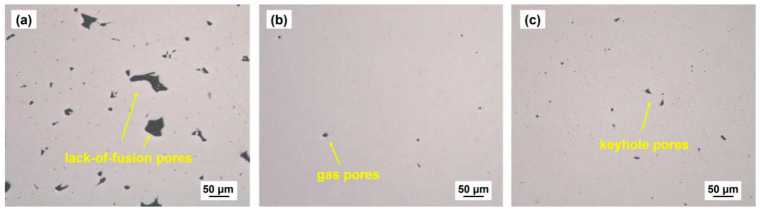
The typical morphologies of internal defects in AM porous structures: (**a**) lack-of-fusion. pores, (**b**) gas pores, and (**c**) keyhole pores [109].

Across the literature, three typical pore morphologies dominate in laser-based additive manufacturing: irregular LoF pores, near-spherical keyhole pores, and gas-induced pores. Collectively, these studies demonstrate that pore size, morphology, and distribution are jointly determined by laser power, scanning speed, and powder morphology. While process parameters vary widely across reports, a universal trend emerges: excessive energy input tends to enlarge keyhole pores, while insufficient energy leads to frequent LoF defects, as shown in Figure 19. LoF defects are among the most detrimental defects in AM. As illustrated in Figure 19a, LoF defects generally exhibit an irregular, flat morphology with sharp edges, and the pores are often filled with unmelted powder particles. The intrinsic mechanism for their formation is insufficient energy input. When the laser power is excessively low, the scanning speed is too high, or the hatch spacing is overly large, the melt pool cannot achieve adequate depth or width to penetrate the previous layer or fuse with adjacent tracks. The risk of LoF defects is particularly high in porous structures or components with fine geometrical features, especially at junctions. LoF defects are the dominant defect controlling fatigue life, as their irregular shape and sharp edges act as severe stress-raisers that directly trigger fatigue crack initiation and early fracture.

The gas pores are generally spherical and small, typically less than 50 μm in size. They mainly originate from two sources: first, inert gas (argon or nitrogen) trapped inside powder particles during the atomization process, which cannot escape within the extremely short melting duration and is thus retained in the solidified material. Second, the protective atmosphere in the build chamber is entrapped by vigorous melt pool flow [107]. During the printing of porous structures, the laser frequently jumps between discrete strut cross-sections, which intensifies melt pool instability. Consequently, the probability of gas pore entrapment is usually higher than that in continuously scanned bulk samples. The keyhole pores form when the laser energy density is excessively high. The recoil pressure induced by metal vaporization depresses the melt pool surface, creating a deep and narrow vapor depression [108]. Once the keyhole becomes unstable and collapses, vapor bubbles at the bottom are trapped by the rapidly advancing solidification front before they can float up, resulting in large irregular or pear-shaped pores with high aspect ratios, as displayed in Figure 19c [109]. Keyhole porosity dominates the degradation of tensile and yield strength, as large, irregular pores reduce the effective bearing area and induce localized damage. For porous structures, especially at nodal regions, overlapping laser scanning paths cause severe local heat accumulation, which readily triggers the keyhole effect. Internal pores act as stress raisers and significantly degrade the fatigue limit and fracture toughness of stress-concentrated regions. In particular, pores located on the surface or at the nodes of struts in porous structures easily act as crack initiation sites, accelerating fatigue failure [110,111]. Additionally, a strong coupling relationship exists between the spatial orientation of the pores and the laser path. This further exacerbates the anisotropy of the mechanical properties in AM components [107].

Habee et al. [112] have investigated the evolution of keyhole porosity in Ti6Al4V thin struts fabricated by SLM with respect to strut diameter and linear energy density. They found that strut diameter plays a significant role in regulating pore formation. When the strut diameter is less than 1.25 mm, the shortened scanning length suppresses the formation of a stable keyhole and thus reduces the retention of gas pores. Under the same high energy input, reducing the strut diameter from 1.25 mm to 0.25 mm decreased porosity by nearly half, whereas strut shape had little influence on pore characteristics. This result indicates that a linear energy density higher than the conventional level for bulk materials can be adopted in the fabrication of fine struts, ensuring sufficient melting while effectively avoiding keyhole porosity. This provides a new pathway for the rational design of process windows for porous lattice structures, which is distinct from the traditional approach to reducing energy density.

### 4.4. Post-Processing Strategies

Post-processing strategies, such as heat treatment, HIP, and surface finishing, are the core regulation bridge in the PSP framework of AM Ni-based superalloy porous structures. Aiming at the non-equilibrium microstructures and typical defects formed by the AM process, post-processing optimizes the microstructure and eliminates defects by applying additional processes, thereby realizing a targeted improvement in macroscopic properties. The selection of post-processing strategies is also matched with the topological design of porous structures, which is considered to be the final progress of the PSP framework.

A major inconsistency in the literature surrounds the applicability of standard heat treatment protocols for AM porous Ni-based superalloys (such as Inconel 718). Several researchers advocate that standard homogenization followed by double aging (developed for cast/wrought alloys) sufficiently restores mechanical properties. However, conflicting reports demonstrate that standard protocols often fail to dissolve the severe elemental segregation unique to the high-thermal-gradient AM process, occasionally even triggering strain-age cracking in complex porous nodes. These discrepancies arise because the initial microstructural state of AM components is highly sensitive to the specific machine architecture, scanning strategy, and volumetric energy density used in different studies. This highlights a critical consensus moving forward: AM porous Ni-based superalloys require bespoke, AM-specific thermal profiles rather than historical wrought standards.

#### 4.4.1. Elimination of Residual Stress

Traditional stress-relief annealing temperatures often refer to the standards for wrought parts, with stress relief typically performed at around 980 °C for Ni-based superalloys. At this temperature, most macroscopic residual stresses can be eliminated through dislocation recovery and climb. However, for AM Ni-based superalloys, this temperature range happens to be near the kinetic nose of δ phase precipitation. The research indicates that microsegregation during the AM process generates numerous nucleation sites. Consequently, the precipitation rate of the δ phase in these Ni-based superalloys is much faster than that in wrought parts, and can form abundantly at grain boundaries within just a few minutes. Although an appropriate amount of δ phase can pin grain boundaries and prevent grain growth, an excessive amount of acicular δ phase will consume Nb in the matrix and weaken the subsequent γ″ strengthening potential, and may induce intergranular brittle fracture [113].

For AM Ni-based porous structures, the residual stress elimination effect is closely related to porous topology: (1) Fine-strut lattice structures have low stiffness, and the residual stress can be released at high-temperature stress-relief annealing without causing structural deformation. (2) TPMS structures with continuous curved surfaces have a uniform stress distribution, and annealing treatment can eliminate 90% of residual stress while avoiding δ phase precipitation, as the uniform temperature field during annealing suppresses the local nucleation of the δ phase. In contrast, disordered porous structures require lower annealing temperatures due to uneven pore distribution, as higher temperatures may lead to pore collapse in stress-concentrated regions. To avoid harmful phase precipitation and initiate microstructural homogenization, recent research tends to adopt higher stress-relief temperatures, typically above 1065 °C. At this temperature, the Laves phase begins to dissolve and the sensitive region for δ phase precipitation is avoided [114]. It should be emphasized that the heat-treatment temperature guideline of above 1065 °C is only applicable to bulk Ni-based superalloy components, and cannot be directly extended to porous components with fine lattice struts or thin-walled porous elements. For porous geometries, the fine struts and thin-walled features exhibit significant size effects and structural sensitivity, and the direct application of bulk heat-treatment logic will lead to thermal deformation, grain coarsening, local overheating, and even structural collapse. Therefore, the heat-treatment parameters for porous Ni-based superalloys must be fine-tuned according to the strut diameter, wall thickness, and porous structural characteristics, and a lower temperature range with a more precise holding time is more appropriate for fine-feature porous components [115].

In addition, controlling thermal deformation during the stress-relief process is particularly critical for porous structures. At high temperatures, the material strength decreases such that a mismatch in thermal expansion coefficients between the substrate and the porous structure or uneven heating may induce new plastic deformation. Zaharia et al. [116] employed a standardized homogenization, solid solution, and aging heat treatment process. This strategy includes the following multi-stage heat treatment procedure: the samples are heated to 1080 °C, held for 1.5 h, and then air-cooled, followed by air-cooling after holding at 980 °C for 1 h, furnace-cooling to 620 °C at 55 °C/h after holding at 720 °C for 8 h, and finally air-cooling after holding at 620 °C for 8 h in sequence. By strictly controlling the heating rate, holding time, and cooling method, this process effectively promoted the dissolution of the Laves phase, the release of Nb elements, and the precipitation of γ′/γ″ strengthening phases, thereby enhancing the microhardness and compressive strength of the material while reducing the tendency toward structural deformation and cracking caused by thermal stress.

#### 4.4.2. Elimination of Non-Equilibrium Detrimental Phases

In AM Ni-based alloys, the as-deposited microstructure typically forms Nb-rich Laves phases, which capture a large amount of the strengthening element Nb and thus negatively affect the alloy performance. The standard AMS 5662 heat treatment strategy (solid solution at 980 °C) is generally insufficient to dissolve these stubborn segregated phases formed during the AM process [117,118]. Liu et al. [119] investigated the Inconel 718 alloy fabricated by SEBM and found that a solid solution treatment at 960 °C could only partially dissolve the Laves phase, while an increase in the solid solution temperature to 1060 °C was required for effective dissolution of the Laves phase. Similarly, Hao et al. [120] studied wire arc AM GH4169 Ni-based superalloy and pointed out that residual Laves phase still existed even after a solid solution treatment at 1020 °C, and a higher solid solution temperature of 1150 °C was needed to completely dissolve the Laves phase.

**Figure 20:**
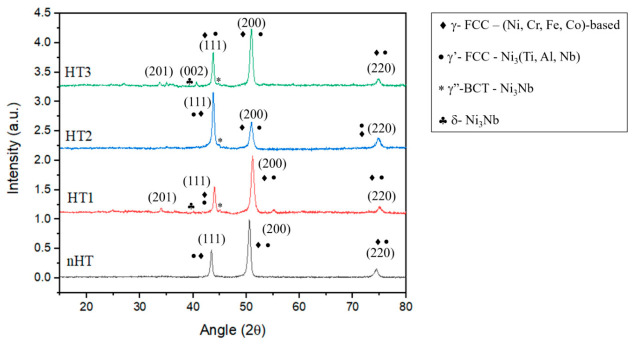
Non-heat-treated and heat-treated IWP samples XRD. The peaks corresponding to the γ matrix, γ″ phase, γ′ phase, and δ phase are indicated [114].

**Figure 21:**
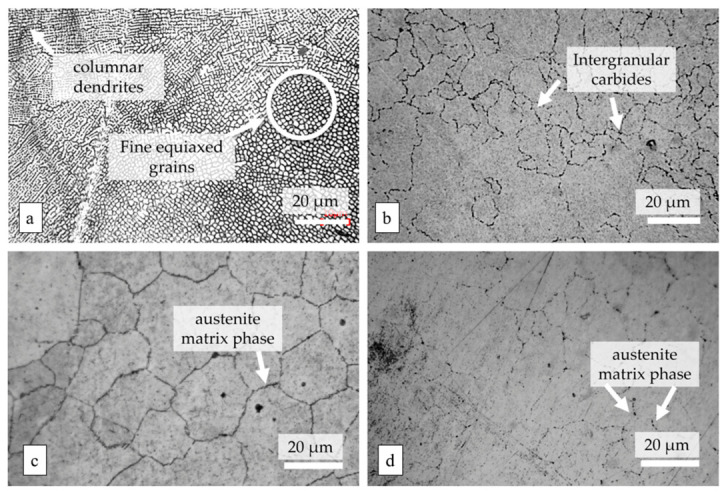
Microstructural analysis of the SLM IN718 subjected to different heat treatments. (**a**) As-built sample (nHT), (**b**) HT1, (**c**) HT2, and (**d**) HT3 [114].

Homogenization heat treatment is a crucial step to eliminate elemental segregation. Its principle is based on solid-state diffusion at high temperatures, which promotes the redistribution of alloying elements throughout the material, thereby reducing compositional inhomogeneity [121]. Homogenization heat treatment typically consists of two stages: the first stage is high-temperature homogenization, involving the long-term annealing of 10~50 h at a temperature slightly below the solidus line to promote the diffusion of elements such as Cr, Nb, and Al, thus effectively eliminating interdendritic segregation. During this process, the heating rate must be controlled to minimize thermal stress and protect the structural integrity of the porous structures. The second stage is aging treatment, which typically adopts a standard two-step aging process. During this stage, fine, dispersed disk-shaped γ″ phases and spherical γ′ phases precipitate from the supersaturated γ matrix. Briones-Montemayor et al. [114] fabricated four types of Inconel 718 lattice structures (BCC, Diamond, Gyroid, and IWP) using SLM and evaluated the effects of three heat treatment strategies of HT1, HT2, and HT3 on their performance. The study found that the HT3 strategy, which combined high-temperature homogenization (1170 °C/1210 °C) with two-step aging (720 °C/621 °C), yielded the most significant improvement in the performance of the lattice structures. The yield strength of the IWP lattice reached 650 MPa, and its energy absorption capacity achieved 40 MJ/m^3^. Compared to HT1 and HT2, HT3 increased the overall yield strength of the lattice structures by approximately 25% and the energy absorption capacity by about 30%. The HT3 process effectively eliminated the residual harmful δ and Laves phases, promoted the uniform and dispersed precipitation of the γ′/γ″ strengthening phases, and reduced the defect density by approximately 40%, as shown in Figure 20 and Figure 21.

Notably, due to their large specific surface area and low heat capacity, the thermal response of porous structures differs significantly from that of bulk materials. During air-cooling, the cooling rate of porous struts may be faster than that of the bulk core, which helps to obtain finer strengthening phases. However, if the Laves phase is not completely dissolved during the solid solution stage, the strengthening effect after aging treatment will be significantly weakened.

#### 4.4.3. Removal of Internal Defects

Internal defects such as pores and cracks are easily generated in AM structures during the fabrication process, which severely affect the mechanical properties and service safety of the components. Hot isostatic pressing (HIP) is a post-processing technology combining high temperature and high pressure, which is widely used to remove internal defects in AM components and serves as an effective method for achieving full densification. Under an argon atmosphere with high temperatures and high pressures, the material undergoes yielding and creep. During the initial pressurization stage, once the local effective stress around the pores exceeds the yield strength of the material at that temperature, the pores will undergo instantaneous plastic collapse. At the pressure-holding stage, residual micropores further shrink and eventually disappear through creep and vacancy diffusion. For cracks or LoF defects, the applied pressure forces the crack surfaces into close contact, and physical welding is achieved through atomic diffusion across the interface, thereby restoring metallic bonds [122,123]. After HIP treatment, the relative density of AM Ni-based superalloys can typically be increased to over 99.9%, the fatigue life can be improved by several times to an order of magnitude, and the ductility is also significantly enhanced. It should be clarified that HIP can increase the relative density to above 99.9% only for fully dense Ni-based superalloy parts. For intentionally porous structures, direct HIP treatment tends to collapse or distort the designed pore architecture, and special strategies must be adopted to maintain a porous morphology.

For AM Ni-based porous structures, the effectiveness of HIP in defect removal depends on pore type and porous topology: (1) Closed gas pores (size < 100 μm) are completely closed by HIP (1160 °C/100 MPa/4 h) for Inconel 718 porous structures, reducing porosity from 2.5% to <0.1%, and the tensile strength is increased by 15~20% [123]. (2) LoF defects (size > 200 μm) in lattice structures require higher pressure and a longer holding time to achieve complete bonding, as the large pores between un-fused struts need sufficient creep deformation to close [119]. (3) Open pores connected to the surface are unaffected by HIP, as the argon atmosphere cannot generate the required pressure gradient for pore collapse—this limits HIP’s effectiveness for high-porosity (≥50%) open-cell structures, where surface-connected pores account for >60% of total porosity [124].

**Figure 22:**
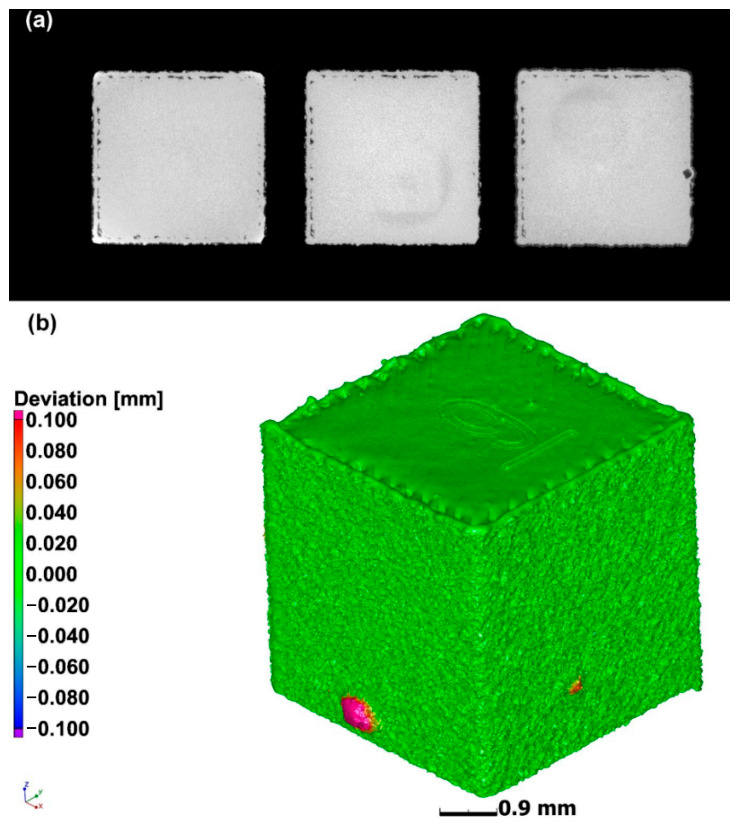
Thermally induced porosity (TIP) blistering observed in porous Ni-based samples after heat treatment. (**a**) Cross-sectional views showing the formation of surface blisters; (**b**) 3D deviation scan visualizing the near-surface blistering caused by trapped gas expansion [124].

Plessis et al. [125] evaluated the pore closure effect of HIP on SLM Ti6Al4V using an X-ray tomography system. The results showed that HIP could effectively close the vast majority of internal pores. The porosity of the optimally processed sample decreased from 0.01% to 0%, the typical LoF porosity reduced from 0.35% to 0.0039%, and even artificial cavities with a diameter of 3 mm were completely densified. However, HIP has a limited effect on surface-connected or near-surface pores. For example, the excessive LoF porosity only decreased from 7.70% to 6.8%, and the contour porosity dropped from 0.911% to 0.643%. The study also found that some closed gas pores would reopen during subsequent heat treatments, and near-surface pores might exhibit a blistering phenomenon, as shown in Figure 22, which is known as “thermally induced porosity (TIP)”. When pores trap the shielding gas involved during the printing process, the high-pressure environment compresses the bubbles to extremely small sizes, making them appear to disappear. In fact, argon atoms have extremely low solubility in the Ni matrix and are difficult to diffuse. During subsequent high-temperature heat treatments, once the external pressure is removed, the high-pressure gas bubbles will re-expand, leading to a rebound in porosity [125,126]. For Ni-based porous structures, TIP can be suppressed by a post-HIP annealing step (1050 °C/2 h + air cooling), which promotes the diffusion of trapped argon atoms along grain boundaries and reduces bubble pressure—this reduces the porosity rebound rate from 30% to <5% [124].

By eliminating defects, HIP can also improve the tensile strength, ductility, fatigue life, and creep performance of Ni-based superalloys [126]. Wu et al. [127] investigated the HIP effect on the stress rupture behavior of a solution-treated single-crystal Ni-based superalloy (DD419) via HIP treatments (1316 °C, 105 MPa, 3 h and 5 h) under 980 °C/250 MPa. The results indicated that the HIP treatment reduced the porosity of the alloy from approximately 0.59% in the solid solution state to 0.001%, and the maximum volume of micropores decreased from about 62,953 μm^3^ to roughly 1014 μm^3^, thereby increasing the stress rupture life of the alloy by 12.7% to 17.6%. More importantly, HIP eliminated the role of micropores as crack initiation sites, shifted the crack initiation locations to the γ/γ′ interfaces, reduced the crack size, and slowed down the crack propagation rate.

In addition, other studies show that HIP can also reduce material anisotropy. Maj et al. [128] investigated the effect of HIP post-processing (1160 °C/100 MPa/4 h) on Hastelloy X samples fabricated by SLM. The results showed that the yield strengths of the as-built XY and XZ orientations were 682 MPa and 621 MPa, respectively, with elongations of 17% and 47%, demonstrating significant directional dependence. After HIP treatment, however, the yield strengths of the two orientations converged (approximately 315~317 MPa), and their elongations became closer (38~41%). Simultaneously, HIP caused the grain size to coarsen from 7.5 μm to 13.5 μm, the texture intensity to decrease from 12.3 times to 3.2 times, and the hardness to drop from 254 HV to 170 HV. This indicates that while HIP effectively enhances material uniformity and ductility, it inevitably causes a decrease in strength due to grain coarsening. For porous structures, this strength loss can be mitigated by combining HIP with aging treatment: after HIP (1160 °C/100 MPa/4 h), Inconel 718 lattice structures are aged at 720 °C/8 h + 620 °C/8 h, which precipitates the fine γ″ phase to compensate for grain coarsening—this results in a yield strength of 520~550 MPa, which is only 5~8% lower than as-deposited samples but with significantly improved ductility and uniformity [125].

To avoid this phenomenon, the modern research trend is to combine HIP with heat treatment. For example, after the high-temperature holding stage of HIP, rapid cooling is performed directly in the HIP furnace, which is used as a quenching furnace to complete the solid solution treatment. Subsequently, aging can be carried out with the same equipment or a conventional furnace. This strategy is not only highly efficient but also prevents grain coarsening during multiple heating cycles, thereby retaining the advantages of fine-grain strengthening [129]. Sommer et al. [130] explored the effects of different sequences of HIP, solid solution treatment, and aging treatment on mechanical properties. The results indicated that HIP effectively eliminated internal pores, increased density, and promoted the subsequent formation of precipitate phases. In addition, the subsequent solid solution treatment further homogenized the microstructure, relieved residual stresses, and significantly improved material hardness and strength by regulating precipitation behavior. Among the tested sequences, the process of HIP followed by solid solution treatment performed best, increasing the ultimate tensile strength to approximately 1165 MPa and the fatigue limit to 210 MPa, which reflects the significant enhancing effect of their synergy on the static and dynamic mechanical properties of the material. Pehlivan et al. [131] evaluated the effects of two post-processing methods, HIP and surface etching (SE), on the structural and mechanical properties of SLM-fabricated rhombic dodecahedron porous titanium alloys. The results showed that although HIP slightly increased the elastic modulus by 5.88%, it had a minimal impact on the overall mechanical behavior of the porous structure and failed to significantly enhance its compressive performance or microstructural integrity. In contrast, SE causes a decrease in stiffness and strength due to strut thinning, but it effectively removes loosely adhered, unmelted powder particles. This significantly reduces the biosafety risks from titanium particle release and brings the structural stiffness closer to that of human bone. Therefore, for orthopedic implant applications, surface etching proved to be a more necessary and practical post-processing method, while the significance of HIP for open-cell porous structures is relatively limited.

#### 4.4.4. Surface Treatment

The extremely high surface roughness of as-built porous structures not only increases the frictional resistance of fluid flow but also makes them more susceptible to fatigue cracks. Due to the complex internal geometry of porous structures, traditional machining cannot reach these inner areas, so it is necessary to rely on fluid-assisted and chemical-assisted surface treatment technologies. Several common surface finishing techniques are as follows:

(1) Abrasive Flow Machining (AFM): AFM is a non-traditional machining technology. When the abrasive medium flows through narrow channels, its velocity increases, enabling the abrasive particles to perform micro-cutting and extrusion grinding on the micro-asperities of internal surfaces [132]. For Ni-based porous structures, AFM is most suitable for truss-based lattice structures with straight struts as the abrasive medium can flow uniformly through the strut channels—this reduces surface roughness from Ra = 8~12 μm to Ra = 1.5~2.5 μm and increases fluid permeability by 20~30% for filtration applications [128]. However, this technology has limitations: the rheological properties of the medium result in directional material removal, and its fluidity is poor in the dead zones of porous structures. In addition, excessive grinding may alter the cross-sectional shape of struts and reduce their diameter, thereby decreasing the overall stiffness of the structure [133].
(2) Shot Peening (SP): SP involves bombarding the part surface with high-speed spherical shots, which induces compressive residual stresses on the surface to enhance fatigue strength and improve surface characteristics [134]. For AM Inconel 718 porous structures, SP with 0.1 mm steel shots at 0.4 MPa pressure reduces surface roughness from Ra = 10~15 μm to Ra = 3~5 μm and introduces a compressive residual stress of 200~300 MPa, which increases the fatigue life by 2~3 times under high-temperature cyclic loading at 600 °C. Research indicates that applying SP to Inconel 718 Ni-based alloy parts fabricated by SLM can improve surface morphology, roughness, waviness, chemical composition, and macroscopic hardness, as well as effectively reducing surface defects [135].
(3) Electropolishing: In electropolishing, the workpiece is used as the anode in an electrolyte solution. Due to the point discharge effect, the current density at micro-asperities is much higher than that in depressions, leading to preferential dissolution of the peaks. This technology is extremely effective in removing adhered powder, passivating the surface, and significantly reducing roughness [136]. Electropolishing not only improves surface finish but also enhances corrosion resistance by forming a dense Cr_2_O_3_ passive film, reducing the corrosion current density by 70~80% in 3.5% NaCl solution. A study found that electropolishing SLM-fabricated Hastelloy X Ni-based superalloy using an eco-friendly deep eutectic solvent (DES) reduced the surface roughness from an initial 10.3 μm to 1.2 μm [137]. However, for components with complex structures, the primary challenge of electropolishing is the uneven current distribution, which easily causes over-polishing in external regions and under-polishing in internal regions [138].
(4) Laser Polishing (LP): LP is a technique that reduces surface roughness by melting and resolidifying the material surface layer, which can effectively reduce Ra from 8~10 μm to 1.0~1.5 μm with minimal material loss and improve the surface properties of additively manufactured metal parts [139]. Research shows that laser polishing can reduce the surface roughness of hardened tool steel from approximately 3.8 μm to below 0.8 μm [140,141].
(5) Surface Mechanical Attrition Treatment (SMAT): SMAT is an advanced surface modification technology. Its core principle is to use high-frequency vibration to cause hard milling balls to repeatedly impact the material surface, introducing severe plastic deformation into the surface layer. This treatment can significantly refine grains, form a nanocrystalline layer, and generate compressive residual stresses, thereby greatly improving the mechanical properties, wear resistance, and corrosion resistance [142,143]. Xu et al. [144] demonstrated that SMAT can induce the formation of a gradient nanostructure on the surface of Ti-Ta layered composites, with the hardened layer thickness reaching up to 9 μm and the surface grain size refined to a minimum of approximately 13.3 nm. Furthermore, both the hardened layer thickness and grain size can be effectively controlled by adjusting process parameters such as feed rate and reduction amount. The combined effects of fine-grain strengthening, dislocation strengthening, and hetero-deformation-induced (HDI) strengthening from this surface nanocrystallization process increase the microhardness to 440 HV and tensile strength to 1097.5 MPa. Simultaneously, the nanocrystalline layer hinders crack propagation through interfaces, leading to a mixed fracture mode of coexisting ductile fracture and quasi-cleavage fracture.

## 5. Properties and Applications of Nickel-Based High-Temperature Porous Structures

This chapter focuses on the mechanical properties and engineering applications of additively manufactured Ni-based superalloy porous structures. The overall performance is synergistically governed by porous architectural topology and manufacturing routes, and clarifying the underlying structure–property relationship is essential for targeted structural optimization and reliable component design. In terms of structural configuration, truss lattices are susceptible to severe stress concentration at nodal connections, while TPMS topologies deliver uniform stress distribution, contributing to superior stiffness efficiency, fatigue resistance, and creep performance under equivalent relative density. By contrast, disordered porous structures present dispersed and unstable mechanical responses due to irregular pore distribution and discontinuous load transfer. Bio-inspired architectures exhibit excellent specific stiffness and energy absorption capacity, yet their comprehensive performance is highly constrained by feature size and hierarchical design strategies.

In terms of additive manufacturing technologies, processing characteristics further differentiate the final microstructure and defect status. SLM produces porous components with refined microstructures and superior tensile strength, but high thermal gradients inevitably induce residual stress and lack-of-fusion defects, significantly compromising fatigue durability. Benefiting from an elevated preheating temperature and stable thermal cycling, SEBM effectively reduces defect density and improves dimensional accuracy, despite its relatively coarser grain microstructures. DED is competent in fabricating large-size porous parts, yet it easily causes severe microstructural inhomogeneity and large-scale defects, restricting its service in fatigue-critical high-performance scenarios. Collectively, structural topology dominates macroscopic stress distribution and load-bearing modes, while manufacturing routes determine microstructural evolution and defect characteristics, which jointly control the tensile, fatigue, and high-temperature mechanical behaviors of porous Ni-based superalloys.

Notably, existing studies reached contradictory conclusions regarding the mechanical size effect of thin struts in such porous systems. Several investigations support a “smaller-is-stronger” tendency, where the rapid solidification of fine struts produces refined cellular microstructures and improves local yield strength. However, numerous studies have demonstrated deteriorated mechanical performance with a reduction in strut dimensions. Such discrepancies stem from the competitive effect between microstructural strengthening and surface defect deterioration. The inherent surface roughness induced by additive manufacturing remains nearly unchanged irrespective of strut size; for ultra-thin struts, the increased roughness-to-thickness ratio acts as a critical structural notch and offsets the beneficial effect of grain refinement. In essence, these inconsistent results are closely related to sample surface state (as-built or post-treated) and manufacturing parameters such as laser spot size, which together determine the competitive relationship between microstructure optimization and defect deterioration.

### 5.1. High-Temperature Mechanical Properties

Investigating the mechanical behavior of Ni-based porous structures in high-temperature environments is essential for lightweight design and structural safety assessments. Unlike room-temperature deformation, which relies primarily on geometric stiffness, high temperatures trigger dynamic metallurgical responses in the matrix material, consequently making the specific strength and energy absorption characteristics of the structure temperature-dependent. For example, Ni-based porous alloys such as Inconel 718 maintain excellent specific strength at elevated temperatures. Research shows that Inconel 718 lattice structures fabricated via SLM exhibit a distinct strengthening peak at 650 °C after proper heat treatment, with the yield strength and compressive strength reaching 540 MPa and 1231 MPa, respectively. This phenomenon is mainly attributed to the dynamic stability of the γ phase and the pinning effect of the δ phase at grain boundaries during high-temperature loading, both of which effectively suppress dislocation glide [145].

**Figure 23:**
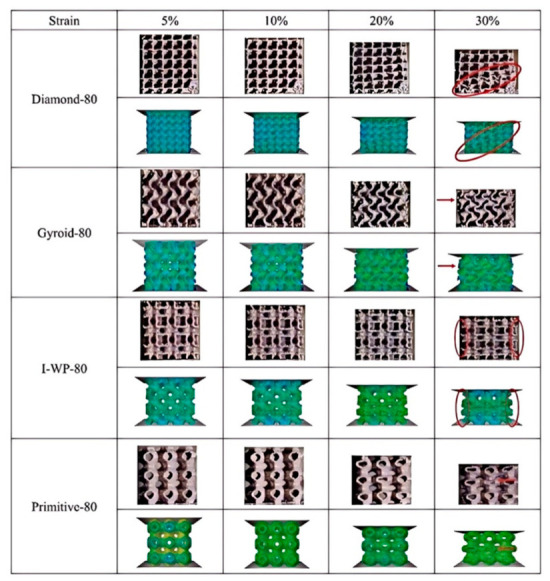
Compressive deformation and stress distribution of four TPMS structures at different strains [140].

As for the topological configurations, TPMS structure, particularly the IWP configuration, demonstrate superior load-bearing stability compared to traditional strut-based lattices during high-temperature compression. Xu et al. [146] evaluated the high-temperature compressive properties and energy absorption of four Inconel 625 TPMS configurations of Primitive, IWP, Diamond, and Gyroid. The results indicate that the IWP structure shows the best overall performance: at 250 °C with 70% porosity, it achieved the highest yield strength of 225.76 MPa and the strongest energy absorption capacity, with a cumulative unit volume energy absorption of 8284.58 MJ/m^3^. In contrast, under the same conditions, the yield strengths of the Diamond, Gyroid, and Primitive structures were 152.42 MPa, 138.18 MPa, and 124.95 MPa, respectively, with energy absorption values decreasing in turn. As shown in Figure 23, deformation mechanism analysis shows that the IWP structure bears loads via uniform stress distribution and layer-by-layer plastic collapse. This avoids the local instability from 45° shear bands (common in diamond structures), enabling the IWP configuration to maintain high structural stability and specific strength at high temperatures.

**Figure 24:**
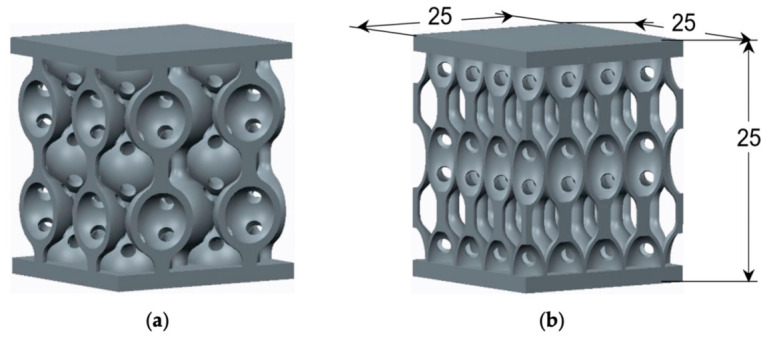
CAD model of lattice structures: (**a**) with spherical stiffening elements and (**b**) with hyperbolic stiffening elements (dimensions in mm) [148].

**Figure 25:**
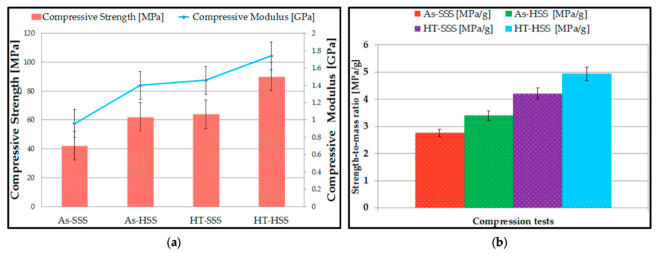
(**a**) Compressive strength and (**b**) strength-to-mass ratio of hyperbolic and spherical stiffening lattice structures [148].

In addition, energy absorption capacity is another critical indicator for evaluating the potential of Ni-based porous alloys in terms of impact protection and energy absorbing components. Increasing temperatures alter the failure modes of these porous structures, thereby influencing their energy absorption efficiency. At room temperature or low temperatures, residual stresses and non-equilibrium phases introduced by AM often lead to the brittle fracture of struts during compression, resulting in severe oscillations in the energy absorption curves. However, when the ambient temperature exceeds 600 °C, the ductility of the matrix material is significantly improved, the influence of the brittle Laves phase is weakened, and structural deformation shifts to be dominated by plastic buckling and folding of the struts. This brittle-to-ductile transition greatly smooths the plateau region of the stress–strain curve and increases the energy absorption density per unit volume [147]. Notably, designs featuring hyperbolic stiffness units exhibit distinct advantages. As shown in Figure 24, Zaharia et al. [148] compared the compressive responses of two lattice configurations and found that hyperbolic units, due to their superior geometric topology, exhibit significantly higher load-bearing capacity than spherical units at the same relative density. Notably, as shown in Figure 25a, the compressive strength of the as-fabricated hyperbolic structure of 62 MPa is 47.6% higher than that of the spherical structure of 42 MPa. After homogenization heat treatment, the strength of the hyperbolic structure is further increased to 90 MPa, maintaining a 40.6% gap compared with the heat-treated spherical structure of 64 MPa. As shown by the integrated area of the load–displacement curves in Figure 25b, the synergy between the hyperbolic configuration and thermal processing increases energy absorption by ~50%. This confirms that the geometric configuration governs load transfer, while heat treatment optimizes matrix strength and toughness.

At high temperatures, Ni-based superalloy undergoes creep, which is a slow plastic deformation over time under constant stress. Creep typically occurs in three stages, initial creep, steady-state creep, and accelerating creep, and the constant strain rate during the steady-state creep stage is a key parameter for predicting the high-temperature service life of materials. For porous alloys, the presence of pores significantly affects their mechanical properties, especially under high-temperature creep conditions. That is because porous structures can reduce the effective load-bearing cross-section of the material and introduce stress concentrations at the pore edges, thereby leading to higher creep rates and shorter creep lives. Internal pores in high-temperature alloy components, such as micropores formed during casting, will seriously threaten their creep resistance [149].

He et al. [150] investigated the evolution of micropores in a single-crystal Ni-based superalloy PWA1483 during creep at 980 °C and 220 MPa. The study found that the number, size, and volume fraction of micropores remain relatively stable during the primary and steady-state creep stages, but increase significantly at the end of the steady-state stage. Specifically, the number of small pores surged from 716 at 101 h to 1476 at 111 h, and the average equivalent diameter of large pores grew from an initial 10.5 μm to 25.57 μm at 115 h. In strain-concentration areas, irregular large pores and high-density micropores are the main creep damage factors, and their rapid growth synchronizes with a higher creep strain rate. Eventually, the surface cracks caused by oxidation connect with internal cracks formed by micropores, leading to creep failure. The research indicates that plastic deformation is the dominant mechanism of creep strain, while the evolution of micropores accelerates the creep damage process.

Currently, research on the creep behavior of Ni-based high-temperature porous alloys mainly focuses on the creep mechanism of the matrix material, the microstructural evolution, and their effects on creep life. However, further in-depth research is still needed regarding the impact of specific porous structures on creep performance, as well as how to improve the creep resistance of porous alloys by optimizing pore structures. Although porous structures are generally regarded as factors that weaken material performance, specific ordered porous configurations may improve the local creep resistance of materials through stress redistribution.

### 5.2. Fatigue Properties

For porous Ni-based superalloy structures serving in demanding high-temperature and high-load environments, fatigue behavior represents a key performance index that determines service reliability. Fatigue failure is predominantly driven by strut-level manufacturing defects, the inherent rough surfaces induced by additive manufacturing, and severe local stress concentrations at strut junctions and nodes. Typical build defects including LoF pores, keyhole porosity, and microcracks act as preferential sites for fatigue crack initiation, while poor surface condition further aggravates stress concentration and accelerates crack propagation. Post-processing strategies such as HIP, chemical polishing and sandblasting can effectively eliminate internal defects, smooth rough surfaces and reduce residual stress, thus remarkably enhancing the fatigue resistance of porous structures. A systematic understanding of these coupled effects provides essential guidance for the anti-fatigue design and engineering application of additively manufactured porous superalloy components. Yamashita et al. [151] applied the √area defect–fatigue limit prediction method proposed by Murakami to the fatigue performance prediction of an SLM-fabricated Ni-based superalloy 718; this method is widely employed for porous structural components. Their results validated that this method can effectively predict the lower bound of the fatigue limit by characterizing the maximum effective defect area, yet the prediction accuracy drops notably for specimens failing due to LoF defects. This deviation arises from the irregular and sharp morphology of LoF defects, which impedes the accurate quantification of effective defect size before fracture. For porous Ni-based superalloy structures, this accuracy reduction is further aggravated by the severe stress localization at strut nodes and thin struts, where LoF defects serve as extreme stress concentration sites that accelerate fatigue crack initiation and propagation. As systematically summarized by Sanaei et al. [152] in their review of defects in additively manufactured metals, internal defects are the dominant contributors to fatigue performance degradation, and their detrimental effects are far more pronounced in porous structures than in bulk materials. This is attributed to the reduced effective load-bearing cross-section and intensified stress concentration in porous architectures, particularly at the geometric discontinuities of strut–node connections. For SLM-fabricated porous Inconel 718, fatigue cracks almost exclusively initiate from defects located at strut junctions or thin-walled regions, and the coupling effect of structural stress concentration and defect-induced stress concentration leads to a sharp decline in fatigue life. A statistical extreme value analysis of defects, as verified in both bulk and porous Inconel 718 components, can effectively quantify the maximum effective defect size within the structural volume, providing a reliable theoretical foundation for fatigue life prediction and the anti-fatigue optimization of additively manufactured porous Ni-based superalloys.

### 5.3. Corrosion Properties

Although Ni-based superalloys possess excellent inherent corrosion resistance, the large specific surface area and unique surface state of AM porous structures complicate their corrosion kinetics in extreme environments. The outstanding corrosion resistance of Ni-based superalloys mainly arises from the formation of a dense passive film in oxidizing media. As an effective diffusion barrier, this film reduces the inward diffusion of oxygen and the outward diffusion of metal ions, thereby protecting the matrix from further corrosion [153].

Alloying elements can further enhance the corrosion resistance of Ni-based superalloys. In this regard, Cr is one of the most critical elements, as it promotes the formation of a stable and dense Cr_2_O_3_ passive film [154]. In addition, Mo stabilizes Cr_2_O_3_ and inhibits the breakdown of the passive film by chloride ions, thus improving the pitting potential of the alloy. Hornu et al. [155] found that the content of alloying elements significantly affects the corrosion behavior of Ni-based superalloys, with Mo contributing the most to the repassivation potential. In a chloride solution at 85 °C, each 1 wt.% increase in Mo raises E_R, CREV_ by approximately 17~18 mV, while Cr and W contribute 5~6 mV/% and ~9 mV/%, respectively. The repassivation ability is maximized at a Cr: Mo: W ratio near 1:3.3:1.65, which is consistent with the coefficients in the Pitting Resistance Equivalent (PRE) formula. This indicates that optimizing the alloy composition is an effective approach to improving the crevice corrosion resistance of Ni-based superalloys in chloride-containing environments. However, the experimental design has certain limitations. The maximum test temperature was only 117 °C, and the corrosive medium was limited to aqueous chloride solutions, which cannot reflect the complex mechanisms such as high-temperature oxidation, hot corrosion, and gas/molten salt corrosion encountered in practical environments like aero-engines. In addition, dense alloy samples were used without considering the effects of porous structures on corrosive medium penetration, local chemical micro-environments, and stress distribution. Future research should extend the corrosion evaluation system to higher temperatures and consider the coupled effects of porous structure, thermal cycling, stress, and multi-component corrosive media. In addition, Chen et al. [156] also found that an appropriate addition of Mo improves the hot corrosion resistance of alloys in NaCl environments. This is mainly attributed to the formation of a dense and protective three-layer oxide film (NiO-NiCr_2_O_4_-Cr_2_O_3_) promoted by Mo, which effectively restricts the diffusion of NaCl and oxygen. Additionally, volatile oxides and chlorides formed by Mo during corrosion create micropores in the oxide film, which release thermal and internal stresses and reduce film cracking. However, excessive Mo generates too many micropores that act as diffusion paths for oxygen, aggravating internal oxidation and weakening the protective effect.

**Figure 26:**
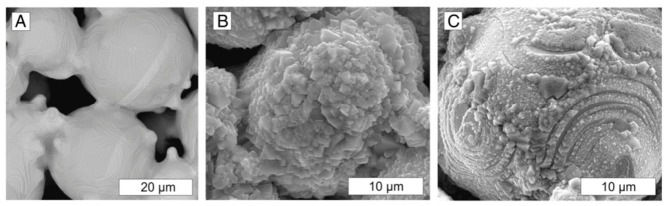
SEM images of not-modified samples oxidized for 1000 h in air at 700 °C: (**A**) Unoxidized sample; (**B**,**C**) Oxidized samples [158].

Porous structures have a dual impact on the corrosion behavior of Ni-based alloys. On the one hand, high porosity and connectivity increase the contact area between the alloy and the corrosive medium, potentially accelerating corrosion. Wu et al. [157] investigated the corrosion behavior of porous Ni_3_Al alloys with porosities ranging from 38% to 60% in 6 mol/L KOH solution. The results showed that the 60% porosity sample exhibited a much higher corrosion current density (2.056 × 10^−4^ A∙cm^−2^) than the 38% sample (8.124 × 10^−5^ A∙cm^−2^). After 480 h of immersion, the mass loss of the high-porosity sample reached 1%, a much higher rate than the 0.05% of the low-porosity sample. However, the corrosion rate is not strictly proportional to the real surface area. With increasing porosity, the pore size distribution, pore shape, and connectivity also change. The increased fraction of large pores of 100~200 μm and uneven pore walls further enhance susceptibility to localized corrosion. Nevertheless, all porous Ni_3_Al samples show excellent overall corrosion resistance in strong alkaline environments. On the other hand, the pore structure can act as a transport pathway for corrosive media, affecting the diffusion and deposition of corrosion products and thus altering the corrosion kinetics. Karczewski et al. [158] studied the corrosion behavior of a porous IN625 alloy with ~25% open porosity at 700~800 °C. The high specific surface area promotes the formation and growth of the oxide layer. As shown in Figure 26, unmodified samples gained about 3.5% in weight after oxidation at 700 °C for 1000 h and almost completely lost their open porosity, indicating that the porous structure accelerates corrosion.

Moreover, rare earth elements, such as Ce, Gd, La, and Y, introduced by impregnation effectively suppress corrosion. Y exhibits the most significant effect, reducing the corrosion rate by nearly 50 times. Under the same conditions, the weight gain is only ~0.5%, and the oxide layer thickness decreases from several micrometers to less than 1 μm. This is because the corrosion of porous alloys is controlled by the outward diffusion of chromium, and the addition of rare earth elements blocks oxygen diffusion and forms stable conductive phases, thereby improving service life in high-temperature oxidizing environments.

Based on the corrosion-resistant properties of Ni-based alloys mentioned above, Ni-based high-temperature porous structures have been widely used in extreme corrosive environments. In the aerospace field, this characteristic ensures the reliable long-term operation of the internal porous cooling structures of turbine blades under high-temperature gas corrosion. In the energy and chemical industry, they can be used for high-temperature flue gas filtration to resist the erosion of acidic and alkaline media and complex atmospheres. Furthermore, in nuclear energy systems and marine equipment, the corrosion resistance of the material ensures the structural integrity and functional durability of porous components under harsh conditions such as chloride ions and irradiation.

### 5.4. Thermal Insulation Properties

Ni-based porous alloys are ideal for high-temperature thermal insulation and thermal barrier systems, as their unique pore structures effectively block continuous heat conduction paths. The effective thermal conductivity decreases exponentially with increasing porosity. For high-porosity lattices, the thermal conductivity is much lower than that of solid structures of the same material [159,160]. This insulation effect comes not only from the low thermal conductivity of air or filler gas, but also from the narrow conduction channels in the metal skeleton. Porous structures reduce the effective cross-section for solid-phase heat transfer. Higher porosity leads to more tortuous heat paths and lower thermal conductivity. Wang et al. [161] showed that pore structure is a key factor governing the effective thermal conductivity of porous insulation materials. Pore shape acts by changing the effective contact area between pores and the matrix. Research indicates that cylindrical pores perpendicular to heat flow provide the best insulation as it has the largest contact area. At 20% porosity, their thermal conductivity is about 5.6% and 2.2% lower than that of spherical and cubic pores, respectively. Pore overlap also affects insulation performance depending on direction: it enhances insulation perpendicular to heat flow but weakens it parallel to the flow. Pore overlap reduces the actual pore volume, increasing thermal conductivity by 4.7~6.2%. In addition, pores suppress gas-phase conduction and convection. Small pores effectively limit gas flow and convective heat transfer. When the pore size reaches the nanoscale, the mean free path of gas molecules exceeds the pore size, triggering the Knudsen effect and further reducing gas thermal conductivity [162,163].

With outstanding high-temperature corrosion resistance, mechanical properties, and thermal insulation capability, Ni-based porous superalloys show broad application prospects in aerospace thermal protection systems. These alloys can be used in extreme high-temperature components such as the leading edges of hypersonic vehicles, combustion chamber liners, and thermal barrier coating substrates for turbine blades, where they withstand extreme temperatures while effectively blocking heat transfer to protect internal structures.

### 5.5. Multi-Functionality of Ni-Based Porous Structures

Porous structures endow Ni-based alloys with unique functionalities. In catalytic reaction engineering, the high thermal conductivity of Ni-based porous alloys makes them ideal supports for strongly exothermic reactions. Compared with conventional ceramic supports, the Ni-based matrix rapidly dissipates reaction heat, effectively eliminating local hot spots in the reactor, preventing catalyst deactivation, and improving product selectivity. Regular pore structures produced by AM provide a large specific surface area for loading active components. Their optimized hydrodynamic design also reduces channeling effects, significantly enhancing conversion efficiency in high-temperature reactions [164].

Beyond their mechanical advantages, additively manufactured porous Ni-based superalloys also demonstrate outstanding functional potential for extreme environmental applications, especially in high-temperature gas filtration [165]. Owing to their unique porous topology and the inherent high-temperature strength of nickel-based superalloys, these porous filters realize stable gas permeation flux and reliable particle retention. They can effectively intercept particles ranging from 20 to 450 nm with a separation efficiency above 99.9%, while enduring severe thermal shock from high-temperature gas back-pulsing at service temperatures up to 1000 °C. Compared with traditional ceramic filters, porous Ni-based superalloy components deliver superior structural robustness, mechanical stability, and regeneration durability, showing great application prospects in aerospace exhaust treatment, coal gasification purification, and nuclear industrial high-temperature gas purification scenarios.

**Figure 27:**
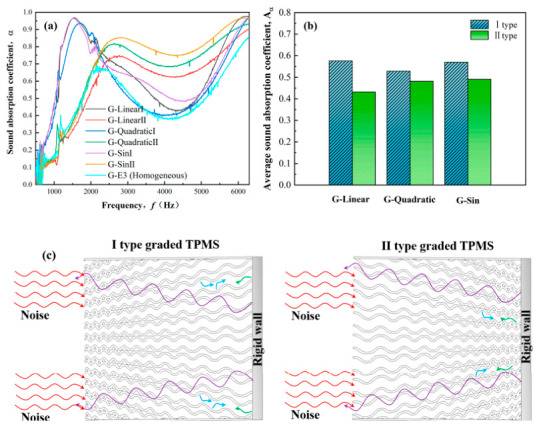
Influence of the function on (**a**) the sound absorption coefficient, (**b**) the average sound absorption coefficient, and (**c**) the mechanism of the sound absorption of graded gyroi [168].

Additionally, porous structures endow Ni-based superalloys with sound absorption and noise reduction capabilities [166]. Porous materials are widely used in acoustic liners of jet engine intake and exhaust ducts to reduce noise [167]. Traditional metal acoustic materials have narrow frequency bands, while Ni-based structures based on TPMS configurations feature interconnected tortuous pores, which increase the viscous dissipation paths of sound waves. Gradient pore structures designed based on the “acoustic black hole” principle can gradually reduce the wave speed and dissipate sound waves after they enter the material. Zhang et al. [168] pointed out that gradient TPMS structures exhibit excellent sound absorption performance. As shown in Figure 27, the Type-I gradient structure with porosity increasing from 60.51% to 77.59% achieves near-perfect sound absorption at 1528 Hz, with a peak coefficient of 0.964. The Type-II structure shows a broader sound absorption frequency band. Furthermore, when the unit cell size of the uniform structure decreases from 4 mm to 2 mm, the first peak sound absorption coefficient increases by 58.97%, and the average sound absorption coefficient increases by 58.18%.

### 5.6. The Difference Between Bulk and Porous Structures

The macroscopic properties and application performance of AM Ni-based superalloy porous structures are inherently the product of a complex coupling among manufacturing processes, molten pool thermodynamics, microstructure evolution, and internal defect distribution. Within the “Process–Structure–Property” framework, the selection of specific AM technologies and post-treatment strategies directly dictates the structural integrity and service reliability of the porous scaffolds. To clarify the influence of mainstream manufacturing schemes, Table 3 systematically compares various processes—including SLM, SEBM, DED, WAAM, and BJ—alongside the “Hot Isostatic Pressing + heat treatment” strategy. This comparison focuses on molten pool stability, microstructural characteristics, typical defects, and final mechanical properties, providing a comprehensive reference for technology selection.

As summarized in Table 3, conventional AM processes have established distinct PSP baselines for Ni-based superalloys. However, treating porous architectures simply as scaled-down bulk materials represents a critical oversight. While substantial progress has been made in optimizing process parameters for dense bulk materials, the fabrication mechanisms and microstructural control for complex porous architectures—particularly at the micro-strut scale—remain insufficiently addressed. The molten pool dynamics within these delicate structures deviate significantly from their bulk counterparts, dictated by varying formation angles, highly constrained strut diameters, and the inherent manufacturing limits of complex topologies. Consequently, current process windows, which are largely developed using standard bulk solid specimens, often fail to account for the intricate requirements of struts with diverse inclinations. This mismatch frequently results in severe lack-of-fusion defects or geometric inaccuracies at topological boundaries.

This discrepancy is further intensified by the distinct thermal histories experienced by struts compared to bulk volumes. The multiscale geometric features of porous scaffolds necessitate unique heat dissipation paths and promote localized thermal accumulation, leading to significant fluctuations in temperature gradients (G) and cooling rates (R). Such variations directly govern microstructural evolution, specifically regarding grain orientation, dendritic spacing, and the degree of elemental segregation. Consequently, these geometry-induced microstructural heterogeneities introduce a high degree of anisotropy and unpredictability in the mechanical performance distribution across the porous network.

This fabrication discrepancy is further intensified by the distinct thermal histories experienced by micro-struts compared to bulk volumes. The multiscale geometric features of porous scaffolds necessitate unique heat dissipation paths. Recent computational fluid dynamics and finite element modeling studies confirm that heat dissipation in thin struts (typically <500 μm in diameter) is heavily restricted to the direction of the previously built layers, acting as a thermal bottleneck. Quantitatively, this geometric restriction causes the temperature gradients (G) to fluctuate violently, often exceeding 10^7^ K/m, while the cooling rates (R) can shift dramatically between 10^4^ and 10^6^ K/s depending on the strut inclination angle relative to the build plate. Such quantified thermal variations directly govern microstructural evolution, fundamentally altering primary cellular dendritic spacing (decreasing from ~1.5 μm in bulk to <0.5 μm in thin struts) and exacerbating the micro-segregation of heavy elements like Nb and Mo.

Finally, this scale-dependent research gap extends directly into the realm of post-processing. The current research gap is further widened by the lack of geometry-dependent post-processing strategies. Standard post-treatment protocols, including heat treatment and Hot Isostatic Pressing, are typically applied to porous structures based on bulk material regimes without considering the kinetic response of micro-scale struts to thermal cycles. Developing synergistic post-processing schemes that simultaneously facilitate defect healing and precise secondary phases (e.g., γ″ and γ′) regulation—specifically tailored to the high surface-to-volume ratios of porous components—remains a pivotal scientific challenge for the engineering application of high-performance Ni-based superalloy structures.

## 6. Conclusions and Outlook

This review represents the first systematic effort to establish a unified process–structure–properties framework for additively manufactured porous Ni-based superalloys. Unlike previous reviews focused on bulk materials or general porous structures, this work emphasizes the unique thermal–mechanical–defect physics that make porous structures an independent material system. The central objective was to fill the gap in systematic understanding of how process parameters, complex topology, microstructure, and defects collectively determine performance. Based on a comprehensive review of the additive manufacturing of porous Ni-based superalloys, the critical scientific and technical challenges identified in the preceding sections can be summarized into four core future directions, which are derived from the synthesis of process, microstructure, defect, and mechanical behavior discussions rather than independent propositions.

(1) Multi-scale modeling from melt pool dynamics to macro-properties must be established.

Current studies show a disconnect between melt pool behavior, solidification kinetics, microstructure evolution, defect formation, and macroscopic mechanical response. A unified multi-scale model that bridges the molten pool scale, microstructure scale, defect scale, and macrostructure scale is urgently needed to enable the quantitative prediction of porosity, grain morphology, phase composition, and defect distribution directly from process parameters.

(2) Precise microstructure control in complex porous topologies must be realized.

As highlighted in the discussion of thermal channel effects and anisotropic grain growth, thin struts and complex curved surfaces in TPMS or bio-inspired structures induce unique thermal histories that differ drastically from bulk materials. Effective strategies must be developed to regulate grain morphology, elemental segregation, and precipitation behavior under spatially varying thermal conditions, especially in nodes and overhang regions.

(3) Reliable fatigue prediction for porous structures must be developed.

Given the dominant role of LoF defects and stress localization in fatigue failure, conventional fatigue models derived from bulk materials are no longer applicable. Future work should establish physics-based fatigue life models that consider defect type, location, size, and stress concentration, particularly at strut joints and thin walls.

(4) AI-driven design must be integrated with physical laws and manufacturing constraints.

As emphasized in the analysis of AI inverse design, purely data-driven methods often produce structures that are physically inconsistent or unmanufacturable. Future design frameworks must embed solid mechanics, heat transfer, and additive manufacturing process constraints into AI models to ensure that the designed porous topologies are structurally stable, thermally reliable, and fabricable.

## Figures and Tables

**Table 1:** Global comparative synthesis of physical mechanisms and mechanical behaviors across different porous structural classes.

Structural Class	Stress Transfer Mechanisms	Geometric Stiffness (Scaling Exponent *n*)	Local Stability and Defect Sensitivity	Deformation Distribution and Energy Absorption
Disordered	Random, tortuous load paths with unpredictable stress peaks	Bending-dominated (*n* ≥ 2); lowest and scattered specific stiffness	Poor. High susceptibility to premature Euler buckling in thin struts	Highly heterogeneous; random local collapse; low energy absorption
Truss-based Lattices	Defined directional paths but severe stress concentration at nodes (*K_t_* > 3.0)	Highly dependent on topology: stretching (*n* ≈ 1) or bending (*n* ≈ 2)	Moderate. Prone to multi-axial localized shearing and fatigue failure at nodes	Localized 45° macroscopic shear bands; abrupt load drops upon failure
TPMS Structures	Continuous, smooth distribution; zero-mean-curvature minimizes peaks (*K_t_* → 1)	Hybrid deformation (1.3 ≤ *n* ≤ 1.6); high specific stiffness and load-bearing	Superior. Continuous surface increases buckling threshold and creep resistance	Homogeneous and global uniform strain; smooth plateau with excellent energy absorption
Bio-inspired	Hierarchical and dynamic stress redistribution across multiple length scales	Spatially tunable (n varies locally); optimized for specific directional loads	High. Tailored local geometries (e.g., gradients) arrest catastrophic failure	Step-wise progressive deformation; controlled energy dissipation via layer sequential collapse

**Table 2:** Systematic hierarchy and prioritization of AM defects across different failure modes in porous Ni-based superalloys.

Dominant Defect Type	Primary Failure Mode Impacted	Underlying Physical Mechanism	Mitigation Strategy
Sharp lack-of-fusion and surface roughness	Fatigue Life	High stress concentration (*K_t_*) acts as pre-existing cracks, accelerating crack propagation and bypassing initiation	Contour parameter optimization; chemical/electrochemical polishing; hot isostatic pressing
Internal gas pores, microcracks and interfacial segregation	Creep Resistance	Promotes creep cavitation and accelerates grain boundary sliding/intergranular fracture at elevated temperatures	Vacuum atmosphere control; HIP to close internal voids; post-AM solution and aging heat treatments
Macro-geometric deviations	Static Strength and Yielding	Reduces effective load-bearing cross-section; converts stretch-dominated struts into bending/buckling-prone elements	Melt pool stability control; in situ monitoring; topology optimization for self-supporting angles
Fine, uniformly distributed spherical micro-pores	Overall Ductility	Minimal stress concentration compared to LoF, but gradually reduces total elongation prior to fracture	Energy density optimization

**Table 3:** Comparison of AM and PSP framework characteristics for nickel-based superalloy porous structures.

Process Parameters	Melt Pool Dynamics	Microstructure	Defects	Final Performance
SLM: High power, fast scan, thin layer	Fast cooling, high thermal gradient	Fine columnar grains, Nb segregation	LoF/keyhole pores, residual stress	High strength, strong anisotropy, defect-sensitive fatigue
SEBM: High preheat, high e-beam energy	Stable melt pool, low thermal gradient	Uniform grains, fine γ′	Low porosity, few hot cracks	Good high-temp. performance, high ductility
DED: High powder feed, large heat input	Slow cooling, severe heat accumulation	Coarse grains, heavy segregation	Interfacial defects, large pores	Low strength, high anisotropy
WAAM: High arc heat, fast deposition	Extremely slow cooling	Coarse columnar grains, δ phase	Hot cracks, warpage	Poor creep, strong anisotropy
BJ: Optimized powder, high-temp. sintering	Solid diffusion, no melting	Uniform grains, no segregation	Sintering pores, low density	Good corrosion, low residual stress
HIP + heat treatment	Pore closure, atomic diffusion	γ″/γ′ precipitation, grain refinement	Defects eliminated, stress relief	Balanced strength-ductility, long fatigue life

## Data Availability

No new data were created or analyzed in this study. Data sharing is not applicable to this article.
